# Supplementary material for: A three-level regulatory mechanism of the aldo-keto reductase subfamily AKR12D
Source: Nat Commun. 2024 Mar 8;15:2128. doi: 10.1038/s41467-024-46363-z (PMC10923870; doi:10.1038/s41467-024-46363-z)
Supplement: Supplementary file 1 — Supplementary Information [file 41467_2024_46363_MOESM1_ESM.pdf]

Supplementary Information

A three-level regulatory mechanism of the aldo-keto reductase subfamily AKR12D

Zhihong Xiao[1], Jinyin Zha[2], Xu Yang[1], Tingting Huang[1], Shuxin Huang[1], Qi Liu[1], Xiaozheng Wang[1], Jie Zhong[2], Jianting Zheng[1], Rubing Liang[1], Zixin Deng[1], Jian Zhang[2]*, Shuangjun Lin[1,3,4]*, Shaobo Dai[1]*

[1]State Key Laboratory of Microbial Metabolism, Joint International Research Laboratory on Metabolic & Developmental Sciences, School of Life Sciences & Biotechnology, Shanghai Jiao Tong University, 800 Dongchuan Road, Shanghai 200240, China

[2]Medicinal Chemistry and Bioinformatics Center, Shanghai Jiao Tong University School of Medicine, Shanghai 200025, China

[3]Haihe Laboratory of Synthetic Biology, Tianjin 300308, China

[4]Frontiers Science Center for Transformative Molecules, Shanghai Jiao Tong University, Shanghai, 200240, China

*Address correspondence to Jian Zhang, Jian.zhang@sjtu.edu.cn, Shuangjun Lin, linsj@sjtu.edu.cn, and Shaobo Dai, sdai@sjtu.edu.cn

Table of Contents:

Supplementary Figures 5

Supplementary Fig 1: Phylogenetic tree analysis of AKRtyl with the annotated AKRs superfamily members. 5

Supplementary Fig 2: A complete sequence similarity network (SSN) of AKR superfamily. 6

Supplementary Fig 3: Multiple sequence alignment of AKRtyl and representative AKRs in each family. 8

Supplementary Fig 4: In *vivo* and in *vitro* characterization of AKRtyl................................ 9

Supplementary Fig 5: SDS-PAGE analysis and size-exclusion chromatography of AKRtyl. 10

Supplementary Fig 6: Determination of the molecular weight of AKRtyl in solution by size-exclusion chromatography coupled with multi-angle light scattering (SEC-MALS).. 11

Supplementary Fig 7: Kinetic curves of AKRtyl with various substrates.......................... 12

Supplementary Fig 8: Kinetic assays of His-tagged and non-His-tagged forms of AKRtyl WT and its mutants.. 13

Supplementary Fig 9: UV-vis spectra measurements of AKRtyl-NADPH complex crystals. 14

Supplementary Fig 10: Overview of AKRtyl structure and cofactor NADPH binding mode. 15

Supplementary Fig 11: Assembly of the AKRtyl octamer and interactions between subunits.. 17

Supplementary Fig 12: Crystal packing analysis of the two purified-state AKRtyl structures. 18

Supplementary Fig 13: The active (R) and dormant (T) conformations with tetramer as the allosteric unit in AKRtyl. 19

Supplementary Fig 14: 2*Fo-Fc* Electron density maps for the active (R) and dormant (T) conformations.. 21

Supplementary Fig 15: Alignment of octamer structures in different liganded states. 23

Supplementary Fig 16: Electron density maps for the inhibitory NADPHs. 24

Supplementary Fig 17: Binding time course of AKRtyl to varying concentrations of NADPH. 25

Supplementary Fig 18: Binding of NADPH to AKRtyl-WT and W331A measured by fluorescence quenching. 26

Supplementary Fig 19: Time course of representative binding of NADPH (25 μM) to AKRtyl-W331A (0.2 μM), indicating one step binding compare to WT........................... 27

Supplementary Fig 20: Kinetics of AKRtyl-W331A to NADPH. 28

Supplementary Fig 21: Conformational selection (the simplest form of the MWC) model and induced fit (the simplest form of the KNF) model fitting for AKRtyl k_{obs1}-[NADPH] data. 29

Supplementary Fig 22: Comparison of NADP (H) binding to AKRtyl and AKR1C9........ 30

Supplementary Fig 23: Surface representation of AKRtyl octamer bound with two tylosins. 31

Supplementary Fig 24: Electron density maps for allosteric tylosins. 32

Supplementary Fig 25: Structural comparison of the tylosin° and the NADPH[i] both bound to the active site. 33

Supplementary Fig 26: Proposed AKRtyl-catalyzed reduction mechanism. 34

Supplementary Fig 27: Kinetics of AKRtyl variants of the orthosteric site........................ 35

Supplementary Fig 28: Kinetics of AKRtyl variants of the allosteric site. 36

Supplementary Fig 29: The single turnover kinetic curves of AKRtyl and its substrate allosteric binding site mutants to tylosin. 37

Supplementary Fig 30: Crystal structure of AKRtyl-tyosin complex without NADP(H)... 38

Supplementary Fig 31: Simulation of AKRtyl-NADP(H) complex with/without allosteric tylosin in the absence of orthosteric substrate. 40

Supplementary Fig 32: Time-independent analysis of conformation populations of monomer models in molecular dynamics. 41

Supplementary Fig 33: Time-independent analysis of conformation populations of octamer models in molecular dynamics. 42

Supplementary Fig 34: Time-independent analysis of volumes of the substrate pockets in monomer models in molecular dynamics. 43

Supplementary Fig 35: Time-independent analysis of volumes of the substrate pockets in octamer models in molecular dynamics. 44

Supplementary Fig 36: The subfamily (AKR12D) represented by AKRtyl is widely distributed in *Streptomyces* with highly conserved features. 45

Supplementary Fig 37: Kinetics of AKR_coelicolor and AKR_albus to NADPH and tylosin. 47

Supplementary Fig 38: Kinetics of other AKRs from three kingdoms of life..................... 48

Supplementary Tables 49

Supplementary Table 1: Activity of AKRtyl on various aldehyde and ketone substrates. . 49

Supplementary Table 2: Comprehensive kinetic data of AKRtyl and its mutants to tylosin in this research. [a].. 50

Supplementary Table 3: Kinetic properties of His-tagged and non-His-tagged enzyme forms of AKRtyl WT and its mutants. 51

Supplementary Table 4: Summary of kinetic properties of AKRs to various ligands in this research.. 52

Supplementary Table 5: Equilibrium constants at different NADPH and tylosin concentrations.. 53

Supplementary Table 6: X-ray crystallographic data collection and refinement statistics.. 54

Supplementary Table 7: Fitting of the fluorescent quenching curves of NADPH to AKRtyl with either double or single exponential model... 56

Supplementary Table 8: Fitting of the fluorescent quenching curves of NADPH to AKRtyl-W331A with either double or single exponential model. 57

Supplementary Table 9: Fit of the equilibrium state fluorescent quenching of NADPH to AKRtyl-WT and 331A with three models.. 58

Supplementary Table 10: Setup of Simulation Systems 59

Supplementary Table 11: Strains and plasmids used in this study.................................... 60

Supplementary Table 12: Primers used in this study. 63

Supplementary References 66

Supplementary Figures

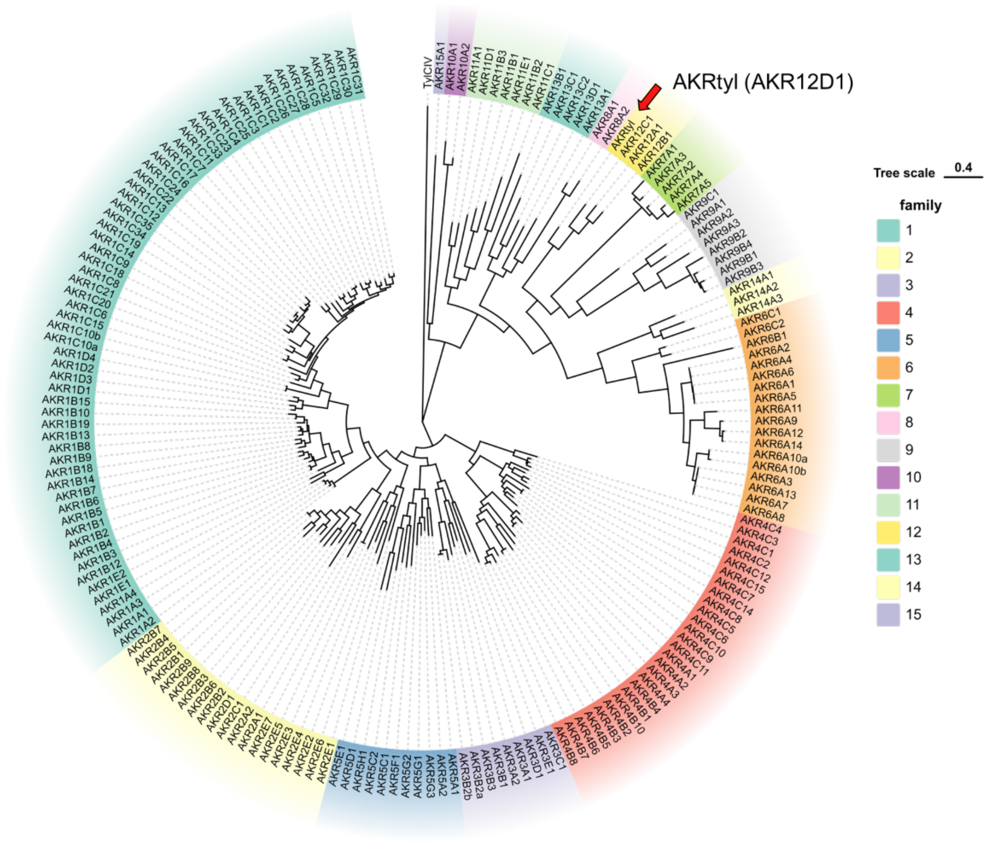

Supplementary Fig 1: Phylogenetic tree analysis of AKRtyl with the annotated AKRs superfamily members.

The AKR sequences were collected from the AKR superfamily database which distributed across 15 families with each distinguished by different color blocks. AKRtyl (red arrow) clusters with AKR12A1 (TylCII), AKR12B1 (EryBII) and AKR12C1 (AveBVII) which are all come from family 12 displayed in yellow color blocks.

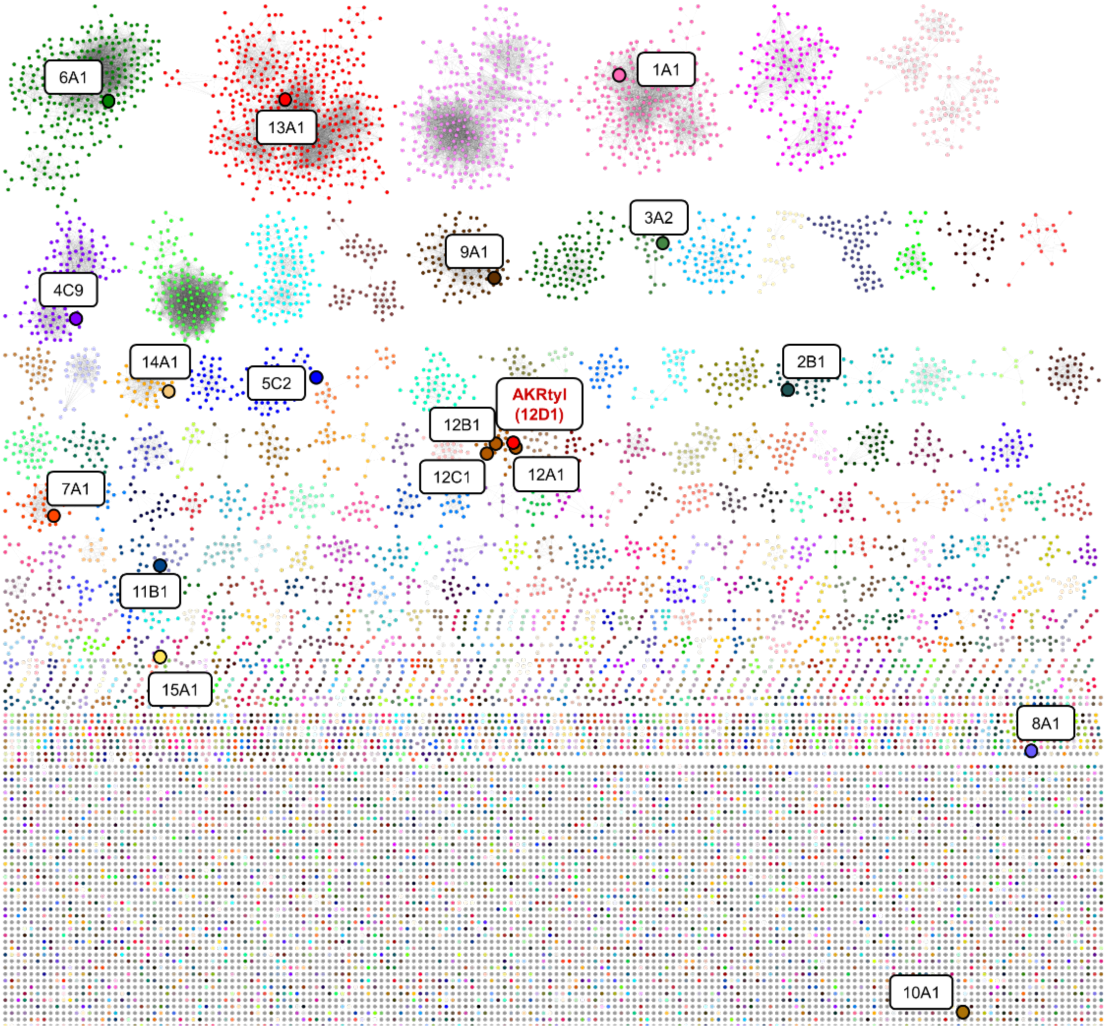

Supplementary Fig 2: A complete sequence similarity network (SSN) of AKR superfamily.

The SSN of over 330,000 sequences from AKR superfamily indicates that they group into many distinct clusters. Sequences were compiled with an alignment score of 88. Each node represents a UniRef50 cluster. Enlarged nodes correspond to sequences with proposed or validated functions and representing each family (the family coloring as shown in Fig. 1b). AKRtyl is labelled in red and clustered with AKR12A1, AKR12B1, AKR12C1.

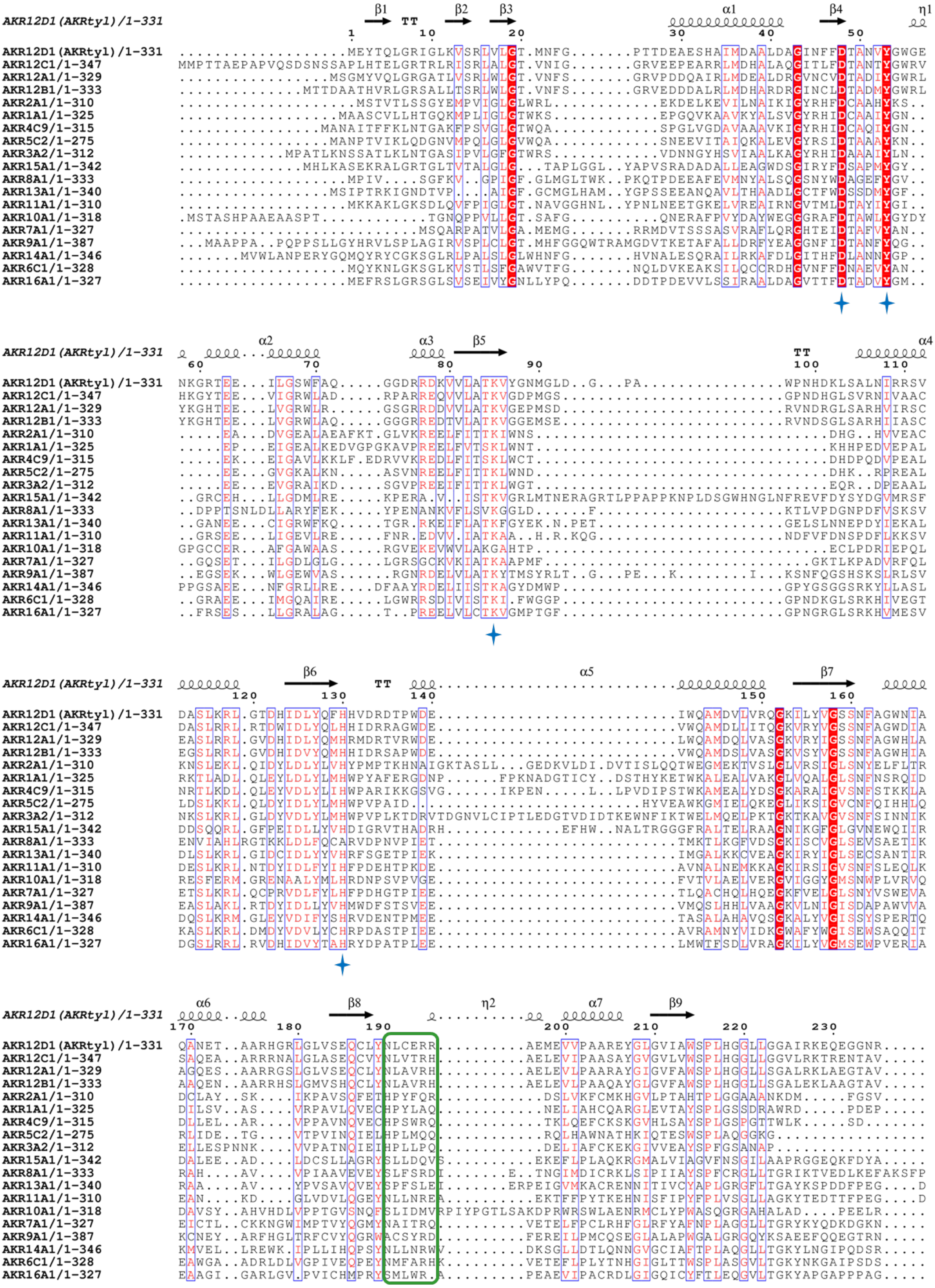

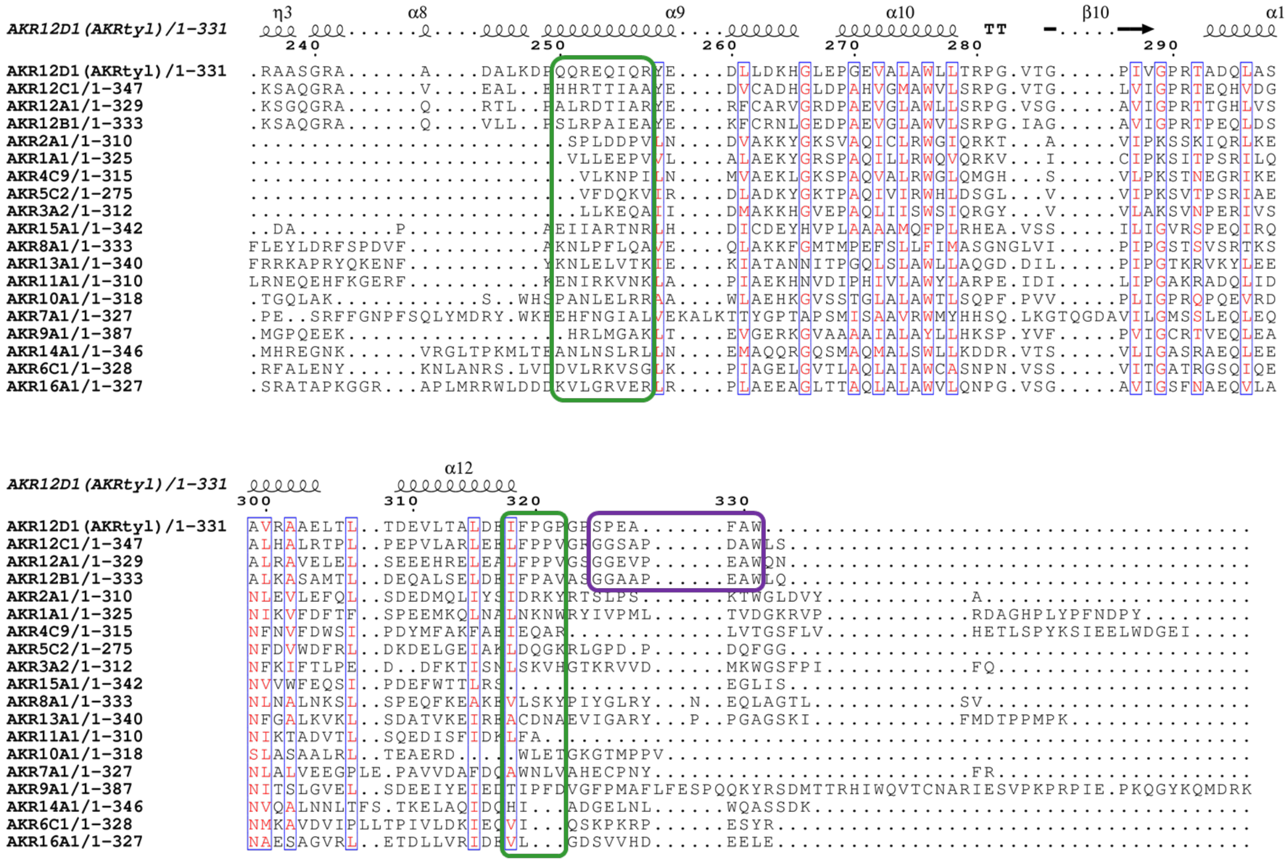

Supplementary Fig 3: Multiple sequence alignment of AKRtyl and representative AKRs in each family.

Amino acid sequences of well-studied AKRs from different families were used for the multiple sequence alignment with AKRtyl. The aligned sequences and their Uniprot accession numbers are: AKR1A1 (*Homo sapiens*, P14550), AKR2A1 (*Malus domestica*, P28475); AKR3A2 (*Saccharomyces cerevisiae*, Q12458); AKR4C9 (*Arabidopsis thaliana*, Q0PGJ6); AKR5C2 (*E. coli*, Q46857); AKR6C1 (*Arabidopsis thaliana*, O23016); AKR7A1 (*Rattus norvegicus*, P38918); AKR8A1 (*Schizosaccharomyces pombe*, O14295); AKR9A1 (*Aspergillus nidulans*, Q00727); AKR10A1 (*Streptomyces bluensis*, AAD28516); AKR11A1 (*Bacillus subtilis*, P46336); AKR12A1 (*Streptomyces fradiae*, A0A3M8EQE1); AKR12B1 (*Saccharopolyspora erythraea*, A4F7P4); AKR12C1 (*Streptomyces avermitilis*, Q79ZL7); AKR13A1 (*Schizosaccharomyces pombe*, Q09923); AKR14A1 (*E.coli*, Q46851); AKR15A1 (*Microbacteriun luteolum*, Q76KC2); AKR16A1 (*Streptomyces tsukubensis*, A0A1V4AAR3). Catalytic tetrads are marked with blue asterisks (D48, Y53, K85, H130 in AKRtyl). The region corresponding to the allosteric tylosin binding site and the C-terminal tail loop in AKRtyl are boxed in green and purple, respective.

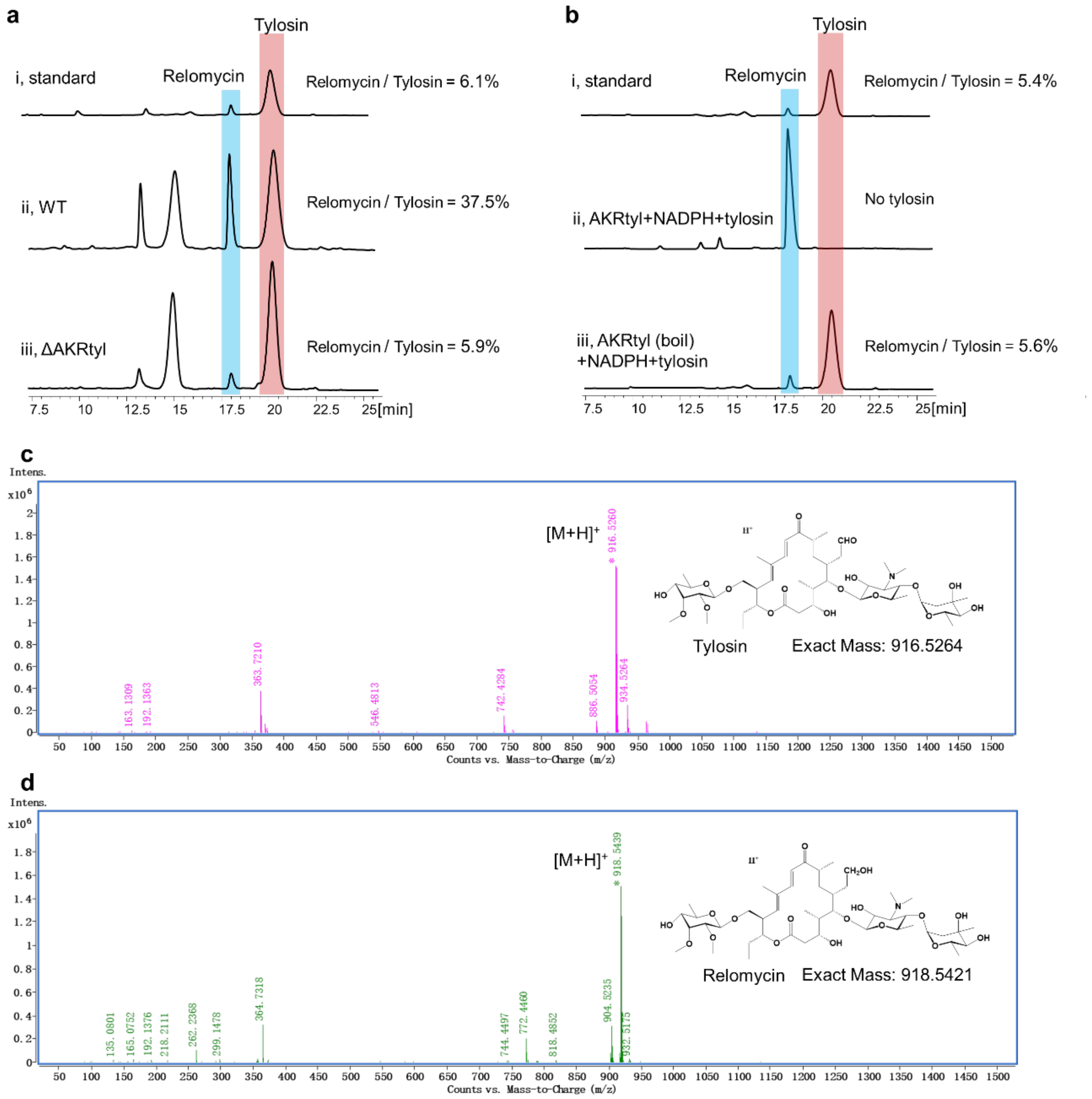

Supplementary Fig 4: In *vivo* and in *vitro* characterization of AKRtyl.

a, HPLC analysis of the fermentation broth of different strains: i) tylosin standard; ii) WT strain; iii) ΔAKRtyl strain. AKRtyl knockout reduces the proportion of relomycin from 37.5% to 5.9%. **b**, HPLC analysis of the enzymatic reactions: i) tylosin standard; ii) AKRtyl (5 μM) + NADPH (1.5 mM) + tylosin (1 mM); iii) AKRtyl (5 μM, boiled) + NADPH (1.5 mM) + tylosin (1 mM). AKRtyl can transform all the tylosin to relomycin. **c**,**d**, High-resolution mass spectrometry analysis (positive ionization mode) of tylosin and relomycin.

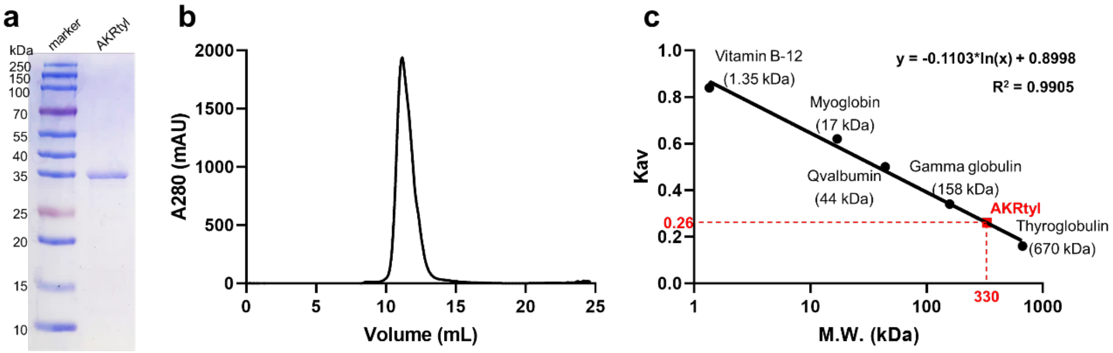

Supplementary Fig 5: SDS-PAGE analysis and size-exclusion chromatography of AKRtyl.

a, The molecular weight of His_6-tagged AKRtyl was approximately 38.4 kDa as calculated from the amino acid sequence, SDS-PAGE analysis showed a smaller molecular size around 35 kDa. **b**, The FPLC profile for AKRtyl (the elution volume is 11.2 ml and the Kav value is 0.26 according to the equation: Kav = $(V_e-V_0)/(V_c-V_0)$); **c.** The calibration curve was generated by using five protein standards, vitamin B-12 (1.35 kDa), Myoglobin (17 kDa), Ovalbumin (44 kDa), Gamma globulin (158 kDa), and Thyroglobulin (670 kDa). The calculated molecule weight of AKRtyl is 330 kDa (Theoretical molecule weight of single strand is about 38.4 kDa), which means AKRtyl is an octamer. AKRtyl was presented as red point, with Kav and calculated molecule weight shown in red.

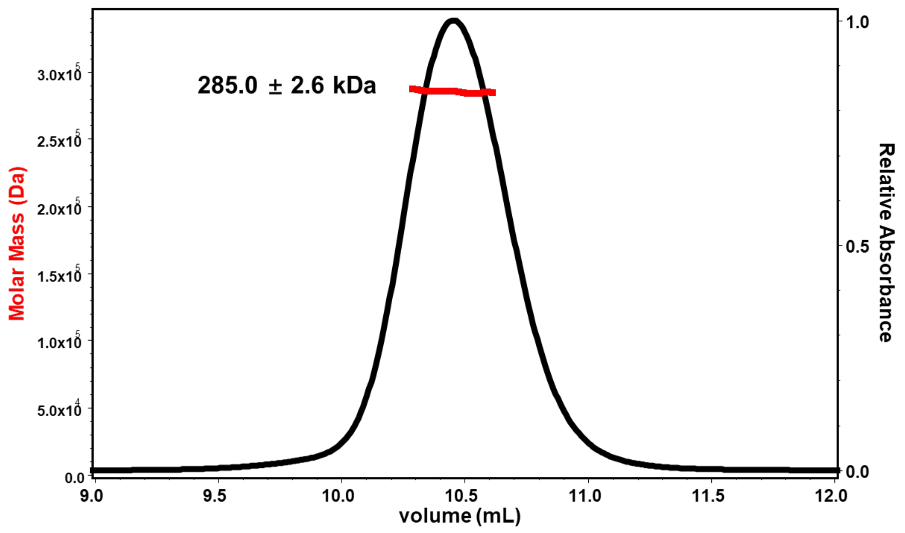

Supplementary Fig 6: Determination of the molecular weight of AKRtyl in solution by size-exclusion chromatography coupled with multi-angle light scattering (SEC-MALS).

The molecular weight of an AKRtyl monomer is 38.4 kDa, and the molecular weight determined in the SEC-MALS experiment is 285.0 kDa which is about 7.5 times of a monomer, so it is concluded that AKRtyl behaves as an octamer in solution.

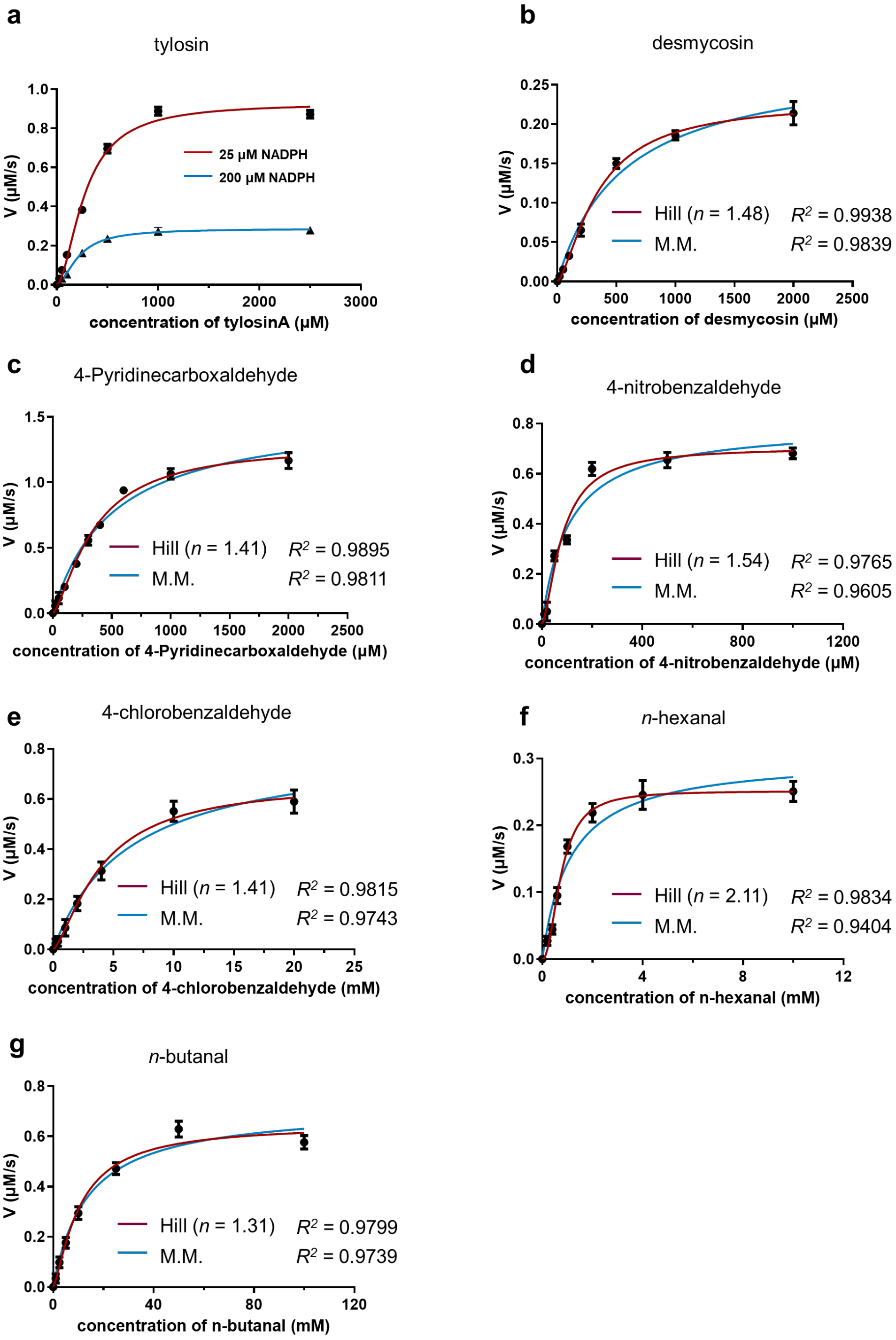

Supplementary Fig 7: Kinetic curves of AKRtyl with various substrates.

a, tylosin, measured at different NADPH concentrations (25 µM and 200 µM); **b,** desmycosin; **c,** 4-pyridinecarboxaldehyde; **d,** 4-nitrobenzaldehyde; **e,** 4-chlorobenzaldehyde; **f,** *n*-hexanal; **g,** *n*-butanal.

Error bars indicate means ± SD (n = 3 independent experiments).

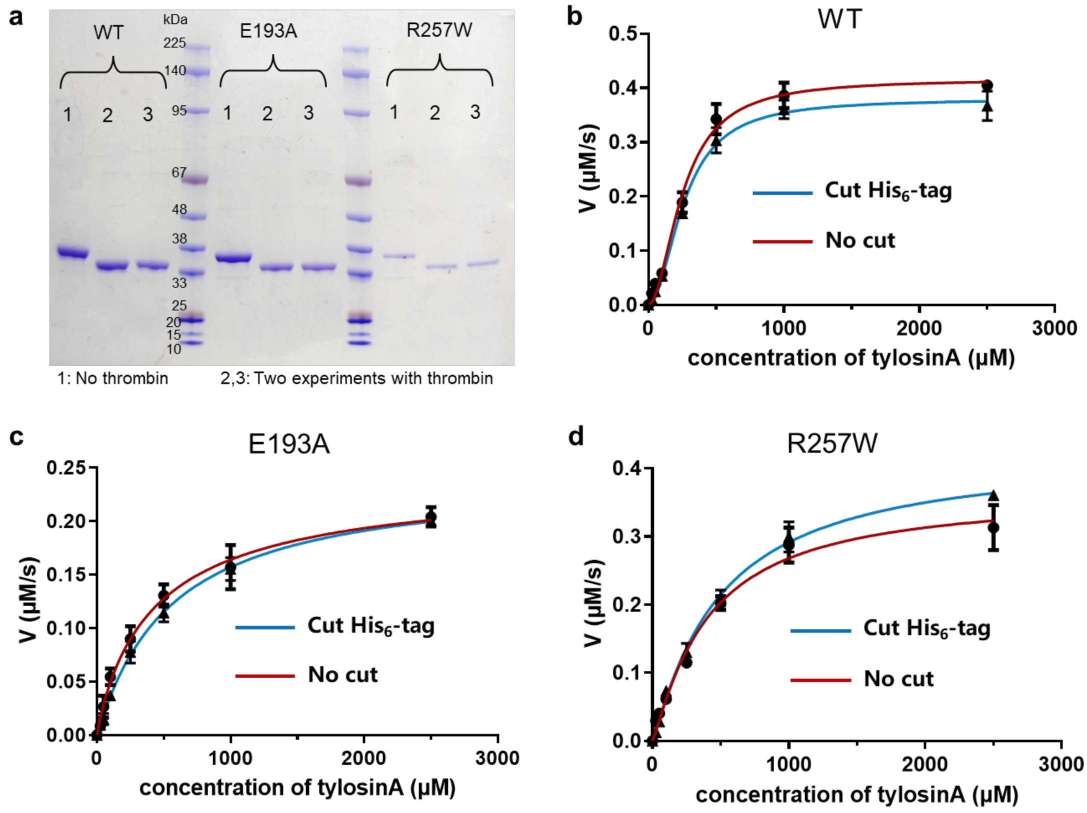

Supplementary Fig 8: Kinetic assays of His-tagged and non-His-tagged forms of AKRtyl WT and its mutants.

a, SDS-PAGE analysis of the His-tag of AKRtyl and its mutants cleaved by thrombin protease (His-tagged: 38.4 kDa; non-His-tagged: 36.3 kDa). **b-d**, Kinetic curves of His-tagged (red line) and non-His-tagged (blue line) forms of AKRtyl WT and its mutants, WT(**b**), E193A(**c**), R257W(**d**). These kinetic data were fitted with the Hill equation. Error bars indicate means ± SD (n = 3 independent experiments).

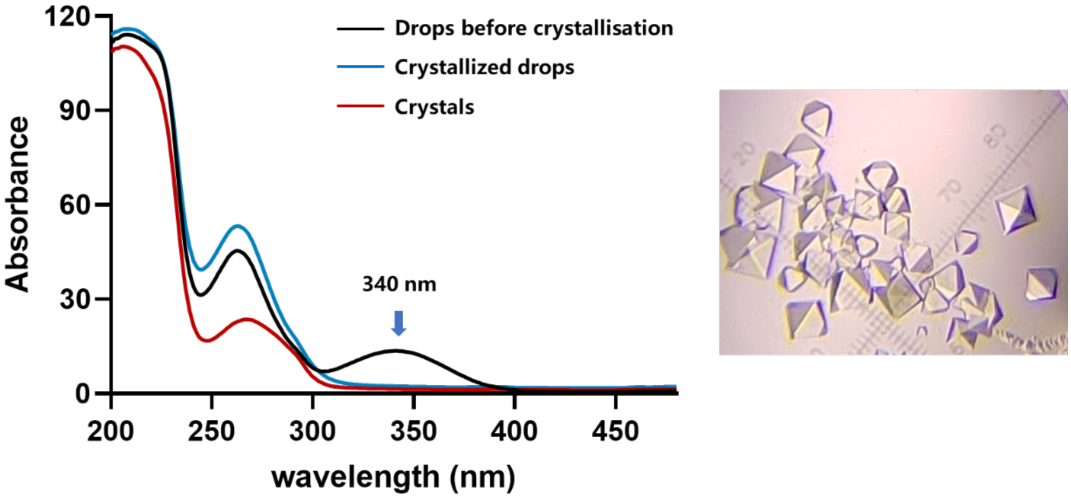

Supplementary Fig 9: UV-vis spectra measurements of AKRtyl-NADPH complex crystals.

There was no 340 nm absorption in either the whole droplet or the crystals.

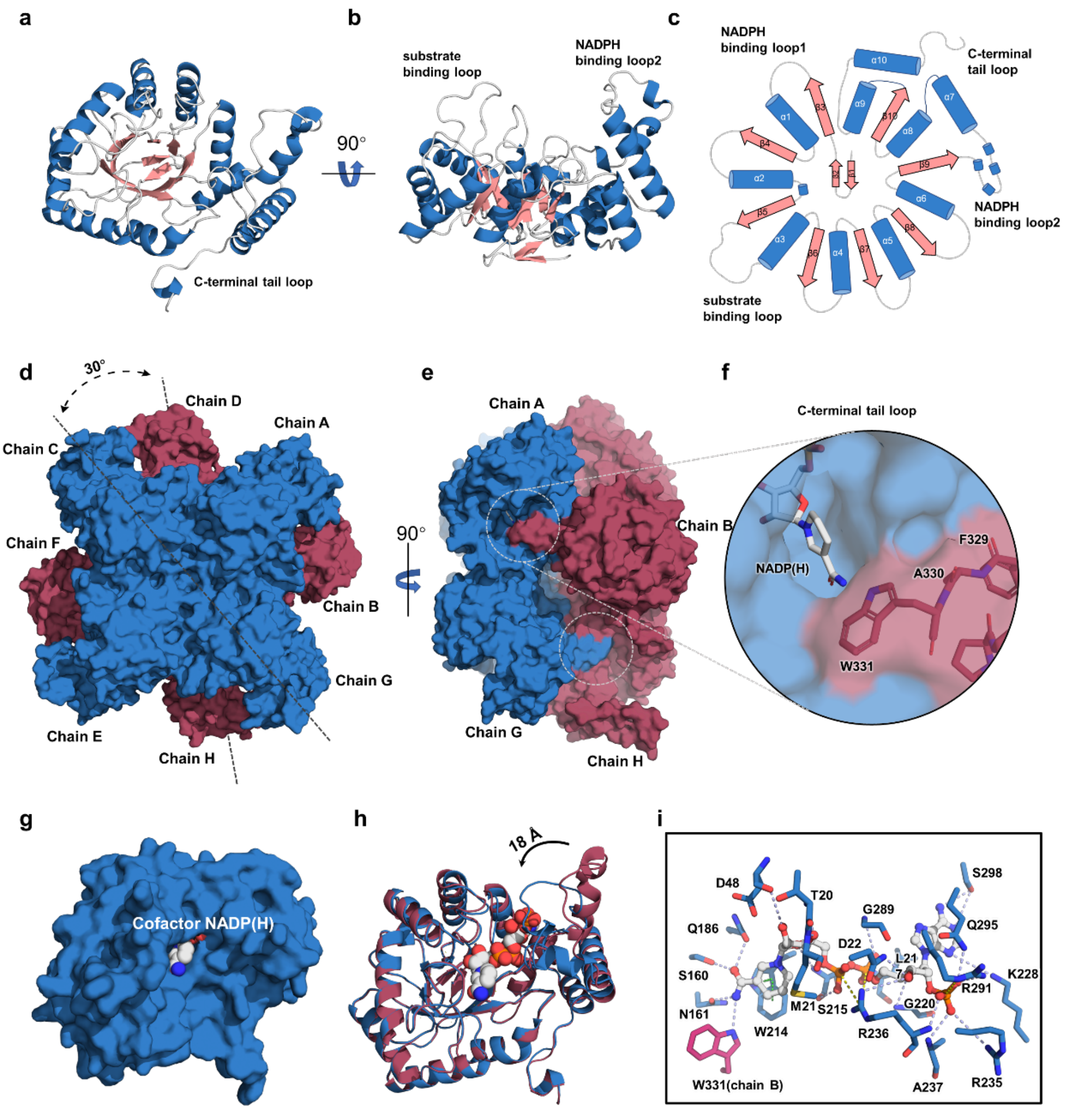

Supplementary Fig 10: Overview of AKRtyl structure and cofactor NADPH binding mode.

a, **b**, Top and side views of AKRtyl monomer (displayed in cartoon mode). **c**, The secondary topology of AKRtyl, α-helices are shown as blue cylinders, β-sheets are shown as pink arrows and loops are shown as gray lines. The structure of AKRtyl monomer consists of a classical $(\alpha/\beta)_8$ fold (TIM barrel). Structural details are as following: a small N-terminal two stranded β-sheets (β1 and β2) capping the TIM barrel, two extra α-helices (α7 and α10) outside of the barrel, and multi-segment loops connecting β-sheets and α-helices, including a substrate binding loop (18 residues) connected β5 and α3, a short cofactor binding loop (loop1, 9 residues) between β3 and

α1, a long cofactor binding loop (loop2, 34 residues) which can also function in substrate binding between β9 and α7 and an unusual C-terminal tail loop (13 residues and the terminal residue is Trp331). **d**, **e**, Top and side views of AKRtyl octamer (displayed in surface mode). AKRtyl adopts an octameric architecture that consists of two well-packed homotetramers enclasped face-to-face with 30° offset where the C-terminal tail loops act as hooks for anchoring. **f**, Close-up of the active center. C-terminal tail loop from the opposite subunit of another tetramer inserts into the active site. **g**, Surface representation of the AKRtyl monomer bound with cofactor NADPH (white spheres). **h**, Superposition of the NADPH bound (blue) and unbound (red) states shows that there is an 18 Å conformational change from an open to a close state upon NADPH binding. **i**, Close-up view of NADPH binding. NADPH extensively interacts with residues inside the cofactor-binding cleft, including hydrogen-bond interactions (light purple dotted lines) and salt bridges (yellow dotted lines). The nicotinamide head ring of NADP(H) is π-π stacked against Trp214 (green dotted lines), and the carboxamide group makes contact with Ser160, Asn161, Gln186, and Trp331 which comes from the C-terminal tail loop of another subunit.

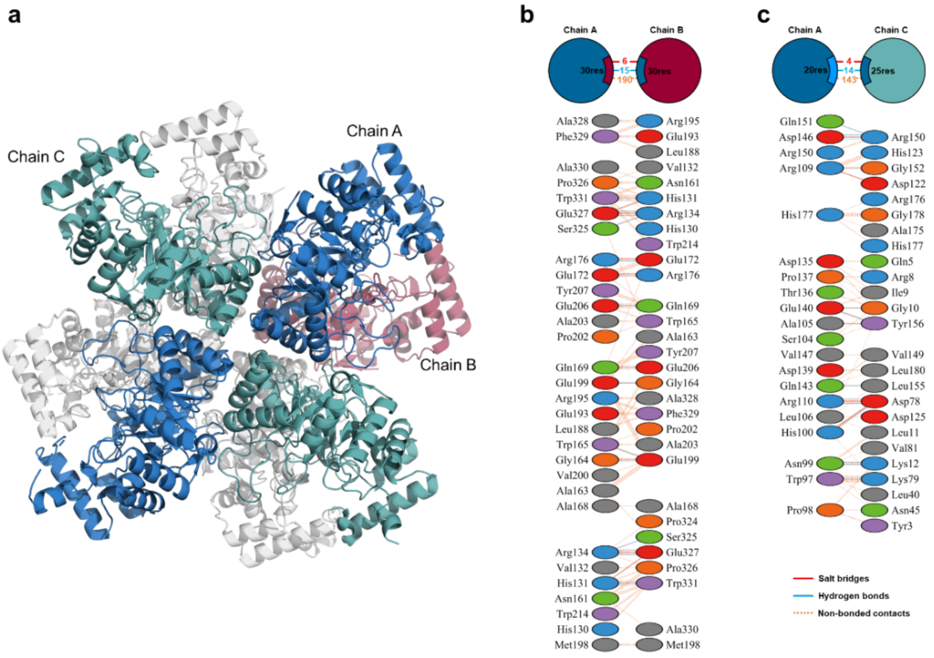

Supplementary Fig 11: Assembly of the AKRtyl octamer and interactions between subunits.

a, The tetrameric assembly in AKRtyl is highly conserved, which is similar as MtmW[1] and other aldo-keto reductases as well as proteins in family 6 that function as voltage-gated K^+ channel β-subunit[2, 3]. The subunits are arranged "end-to-side" through the N-terminal β-sheets at the bottom of the TIM barrel packed against two α-helices (α3 and α4) and the substrate binding loop from an adjacent subunit (blue chains to green chains). This assembly pattern makes each tetramer shaped like a "pinwheel" and the two helices lying outside of the TIM barrels are located on the periphery of the octameric scaffold. **b**, Interactions between two opposite subunits (chain A and chain B) originating from different tetramers involve 6 salt bridges (red), 15 hydrogen bonds (blue) and 190 non-bonded contacts (yellow), burying ~1500 $Å^2$ of the solvent-accessible surface area covered of each subunit. **c**, Interactions of two adjacent subunits (chain A and chain C) of the same tetramer involve 4 salt bridges (red), 14 hydrogen bonds (blue) and 143 non-bonded contacts (yellow), burying ~1100 $Å^2$ of the solvent-accessible surface area of each subunit. The interactions were analyzed by LigPlot+[4] which is incorporated into the PDBsum database[5].

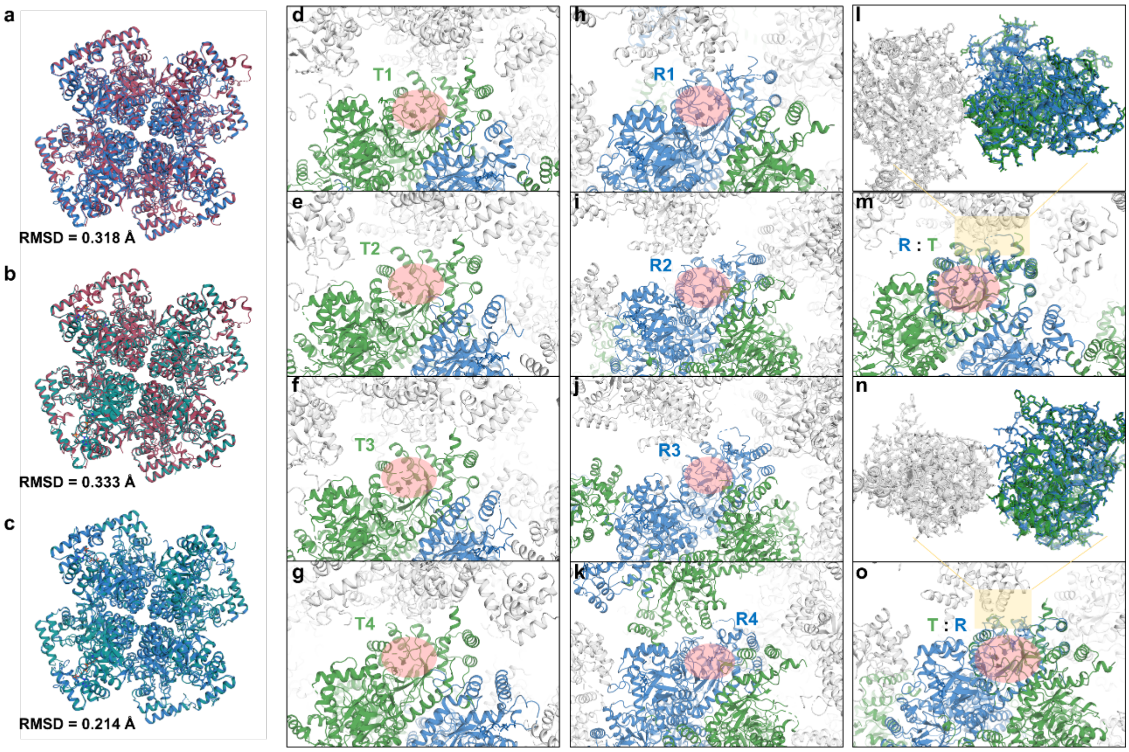

Supplementary Fig 12: Crystal packing analysis of the two purified-state AKRtyl structures.

a-c, One-to-one alignment of three octamers in the two purified-state structures. The octamer configurations are highly consistent. **d-g**, Interactions of each subunit of the dormant conformational tetramer with neighboring asymmetric units. **h-k**, Interactions of each subunit of the active conformational tetramer with neighboring asymmetric units. The dormant conformation is colored green, the active conformation is colored blue, the neighboring asymmetric units are colored grey-white and the structural regions used to distinguish between R and T are shown as transparent ellipse colored in pink. The region used to distinguish between R and T in all the subunits was not occupied or squeezed by the neighboring asymmetric units (shown as cartoon colored in grey-white). **l-o**, Alignment of subunits with one conformation to another conformation (**m**, R align to T; **n**, T align to R). The junction with neighboring asymmetric units is shown as rectangle colored in yellow. Both the R and T conformations are well accommodated in their new positions and do not conflict with other asymmetric units at the junction.

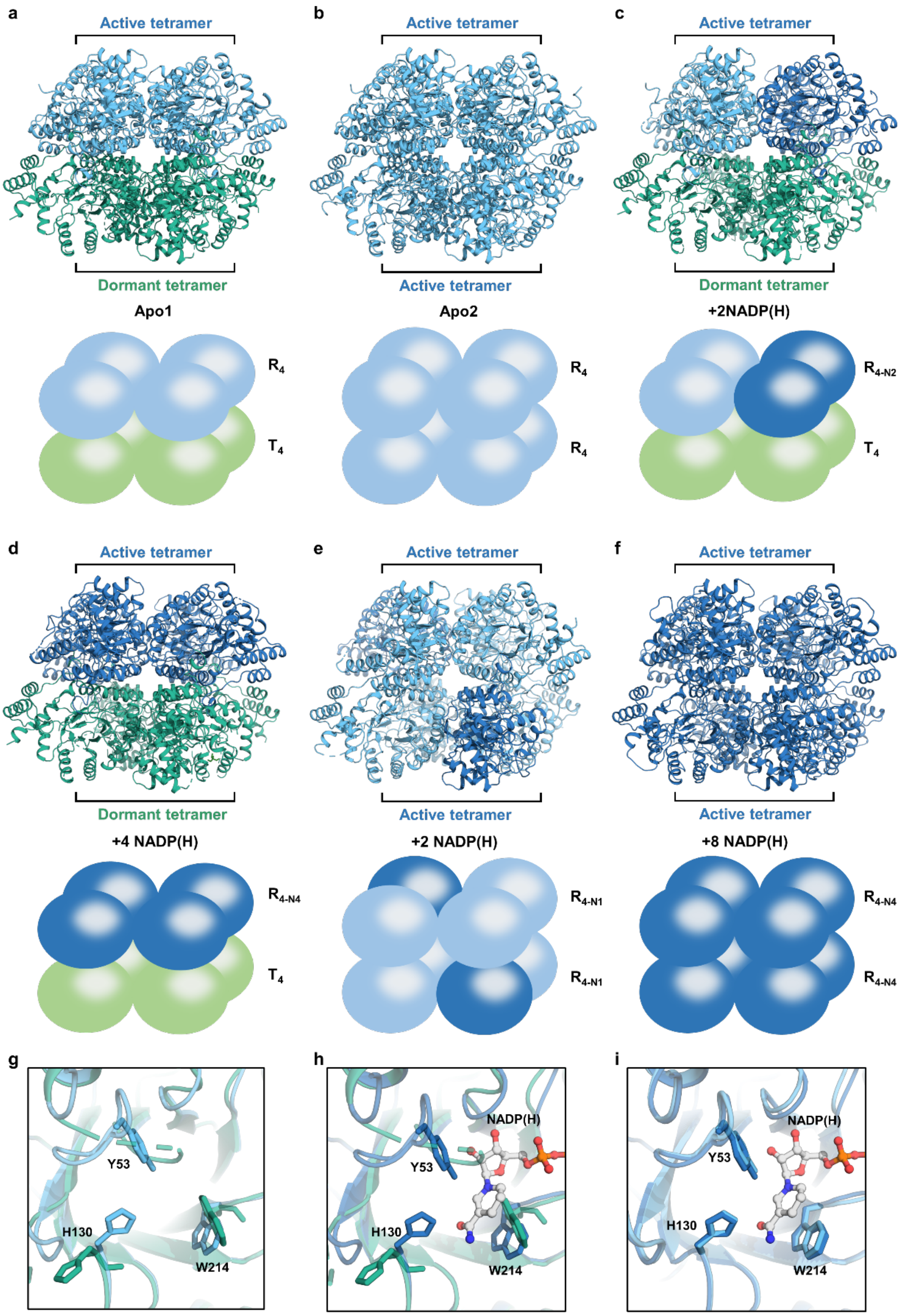

Supplementary Fig 13: The active (R) and dormant (T) conformations with tetramer as the allosteric unit in AKRtyl.

a-f, Cartoon (top) and conformational pattern (bottom) of AKRtyl structures in 6

different conformations and different NADP(H) liganded states. Active subunits (R) are colored light blue, NADP(H) bound dark blue, dormant subunits (T) are colored green. **a**, the first apo structure (R_4T_4 octamer). **b**, the second apo structure (R_4R_4 octamer). **c**, AKRtyl bound 2 NADP(H)s in the same tetramer ($R_{4\text{-}N2}T_4$ octamer). **d**, AKRtyl bound 4 NADP(H)s in the same tetramer ($R_{4\text{-}N4}T_4$ octamer). **e**, AKRtyl bound 1 NADP(H) each in different tetramers ($R_{4\text{-}N1}R_{4\text{-}N1}$ octamer). **f**, AKRtyl bound 8 NADP(H)s in different tetramers ($R_{4\text{-}N4}R_{4\text{-}N4}$ octamer). **g**, Comparison of the active site of the active (light blue) and dormant (green) conformations. In the dormant conformation, the catalytic residue Tyr53 is disordered, the side chain of the catalytic residue His130, faces the opposite direction of the active center, and the side chain of Trp214 twists. **h**, Comparison of the active site of the NADPH-bound active (dark blue, NADPH is shown as white ball-and-stick) and dormant (green) conformations. The twisted Trp214 is not suitable for NADPH binding in the dormant conformation. **i**, Comparison of the active site of the active (light blue) and NADPH-bound active (dark blue) conformations. The NADPH binding residues Trp214, catalytic residues Tyr53 and His130 are ordered and aligned correctly in the active conformation.

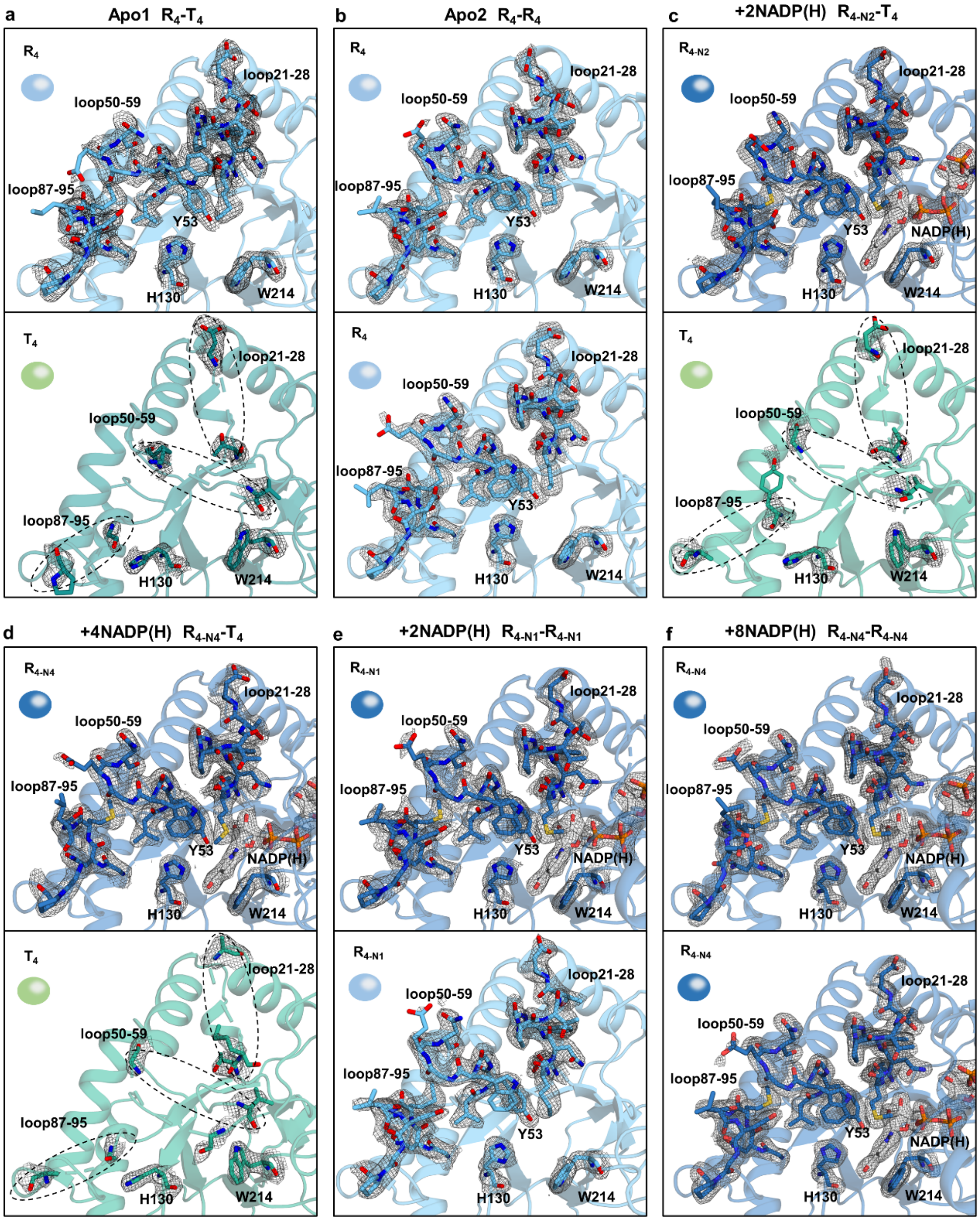

Supplementary Fig 14: 2*Fo-Fc* Electron density maps for the active (R) and dormant (T) conformations.

The maps (**a-f**) correspond to structures (**a-f**) in Supplementary Fig 12 and are contoured at 1.0σ. Each tetramer is shown with the electron density at the difference between the R and T conformations of a representative subunit. Active subunits (R) are colored light blue, NADP(H) bound dark blue, dormant subunits (T) are colored

green. The R conformations with ordered functional loops (loop21-28, 50-59, 87-95) and the cofactor binding pocket (Y53, H130, W214) is clear but the T monomer with functional loops disorder and the cofactor binding pocket is blurred. **a**, the first apo structure (R_4T_4 octamer). **b**, the second apo structure (R_4R_4 octamer). **c**, AKRtyl bound 2 NADP(H)s in the same tetramer ($R_{4\text{-}N2}T_4$ octamer). **d**, AKRtyl bound 4 NADP(H)s in the same tetramer ($R_{4\text{-}N4}T_4$ octamer). **e**, AKRtyl bound 1 NADP(H) each in different tetramers ($R_{4\text{-}N1}R_{4\text{-}N1}$ octamer). **f**, AKRtyl bound 8 NADP(H)s in different tetramers ($R_{4\text{-}N4}R_{4\text{-}N4}$ octamer).

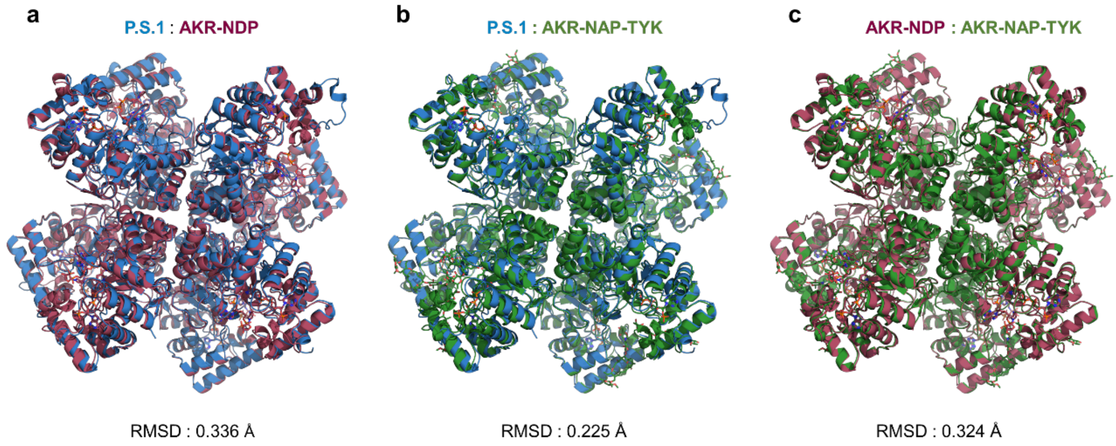

Supplementary Fig 15: Alignment of octamer structures in different liganded states.

a, Alignment of the first purified state (blue, abbreviate as P.S.1) octameric structure with AKRtyl-NADPH octameric structure (red, abbreviate as AKR-NDP) shows a C_α-RMSD of 0.336 Å. **b**, Alignment of the first purified state (blue) octameric structure with AKRtyl-NADP$^+$-tylosin octameric structure (green, abbreviate as AKR-NAP-TYK) shows a C_α-RMSD of 0.225 Å. **c**, Alignment of AKRtyl-NADPH octameric structure (red) with AKRtyl-NADPH octameric structure (red) shows a C_α-RMSD of 0.324 Å.

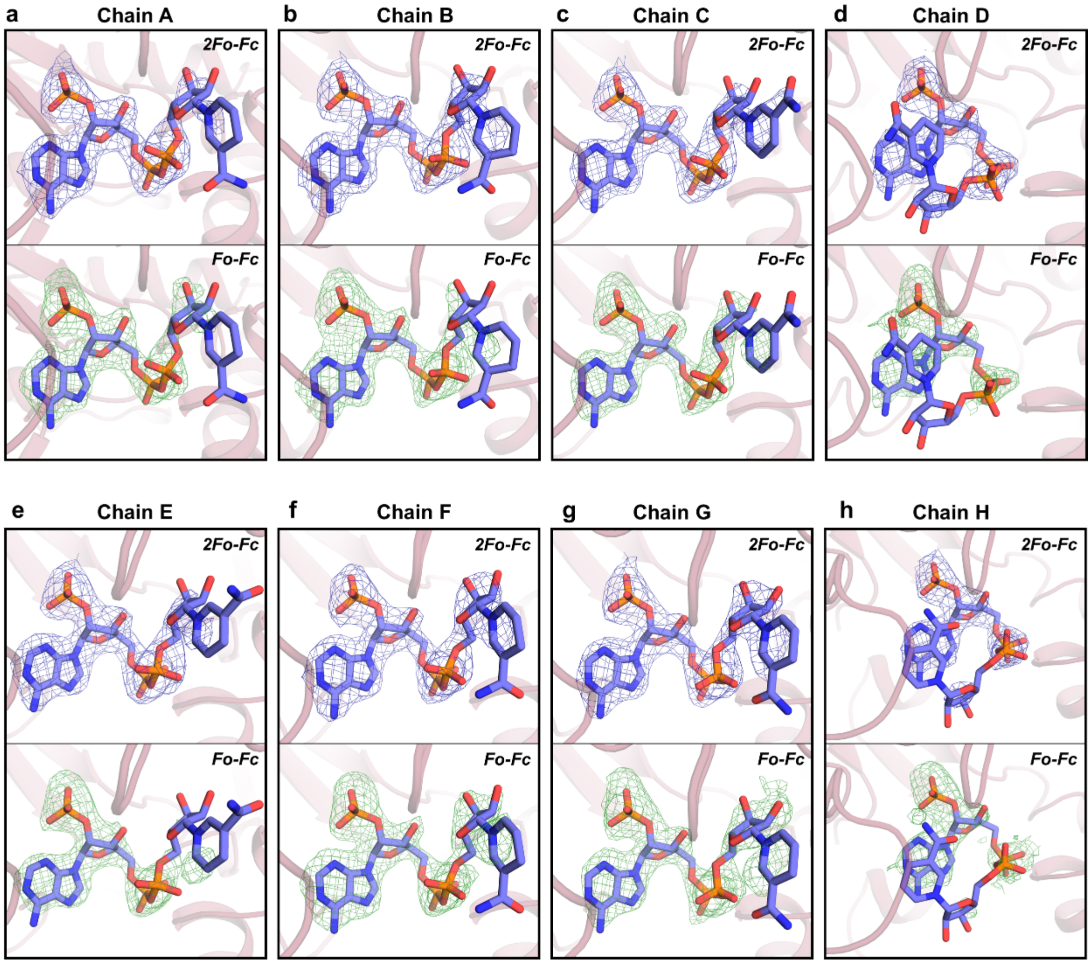

Supplementary Fig 16: Electron density maps for the inhibitory NADPHs.

$2F_o$-F_c electron density maps (shown in blue) for the inhibitory NADPHs in eight chains (up) contoured at 1.0σ; F_o-F_c omit maps (shown in green) for the inhibitory NADPHs in eight chains (down) contoured at 3.0σ. (PDB: 8JWN)

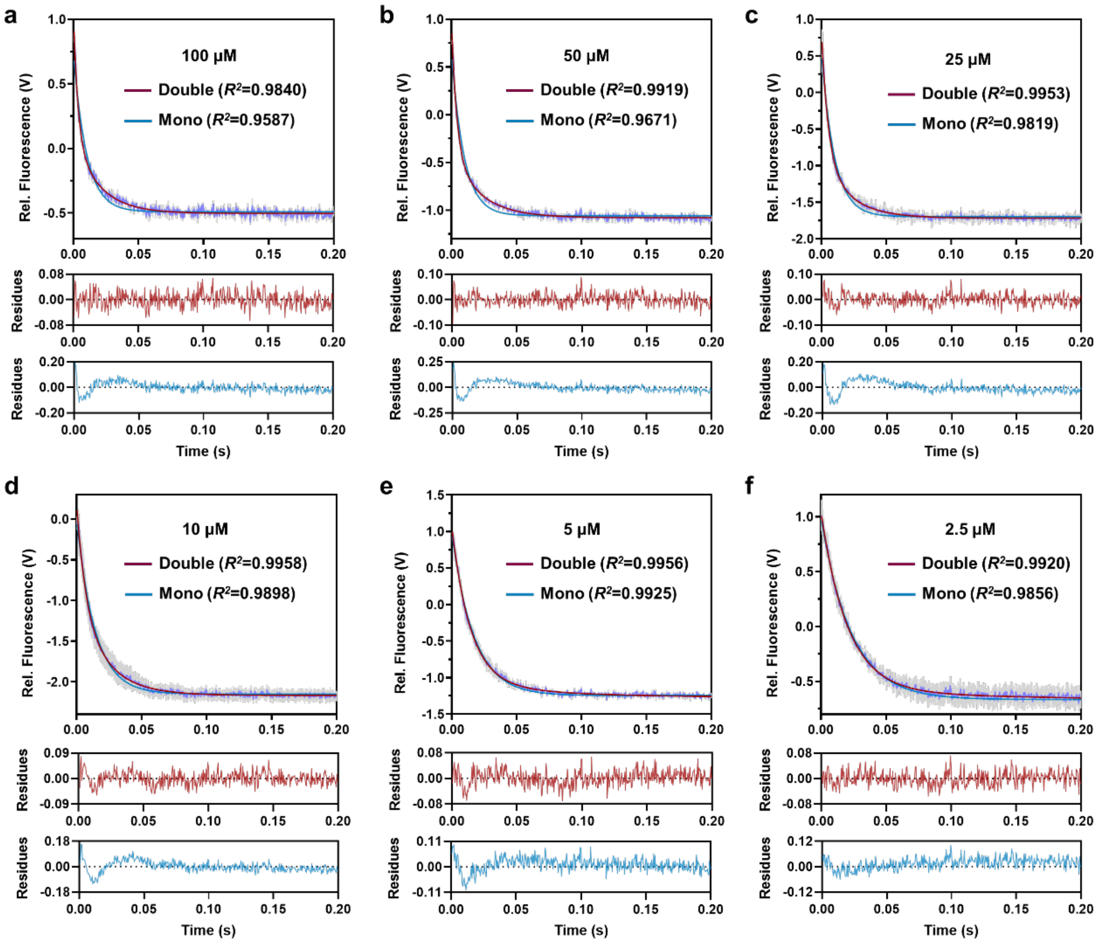

Supplementary Fig 17: Binding time course of AKRtyl to varying concentrations of NADPH.

Time course for binding NADPH (2.5-100 μM) to AKRtyl (0.2 μM) measured by monitoring the tryptophan fluorescence change quenched by NADPH. In the time course curve for each concentration, the purple line represents the average of three consecutive trials and the gray area represents the SD (n = 3 independent experiments). The red line represents the fit of the data with a double-exponential decay and the blue line represents the fit of the data with a mono-exponential decay. The residuals are shown below the fluorescence transient curve.

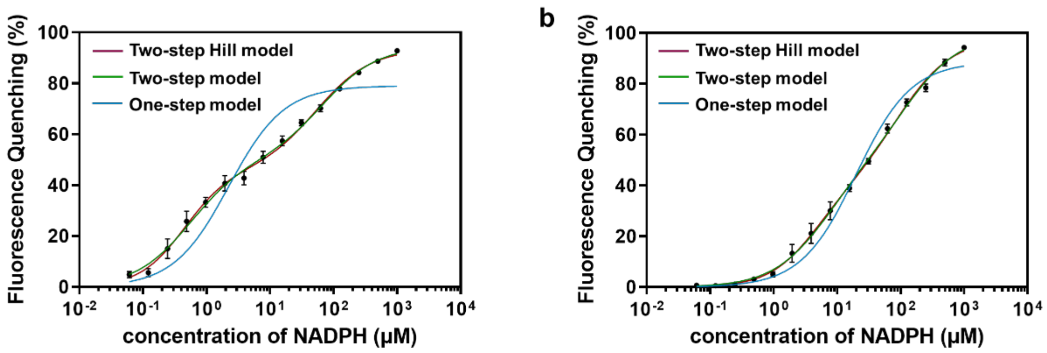

Supplementary Fig 18: Binding of NADPH to AKRtyl-WT and W331A measured by fluorescence quenching.

a, The fluorescence quenching ratio of AKRtyl at varying NADPH concentration at equilibrium. The data were fitted with one-step model (blue line; R^2 = 0.8783), two-step model (green line; R^2 = 0.9587) and two-step Hill model (red line; R^2 = 0. 0.9591). Error bars indicate means ± SD (n = 8 independent experiments). **b**, The fluorescence quenching ratio of AKRtyl-W331A at varying NADPH concentration at equilibrium. The data were fitted with one-step model (blue line; R^2 = 0.9639), two-step model (green line; R^2 = 0.9762) and two-step Hill model (red line; R^2 = 0.9766). Error bars indicate means ± SD (n = 8 independent experiments).

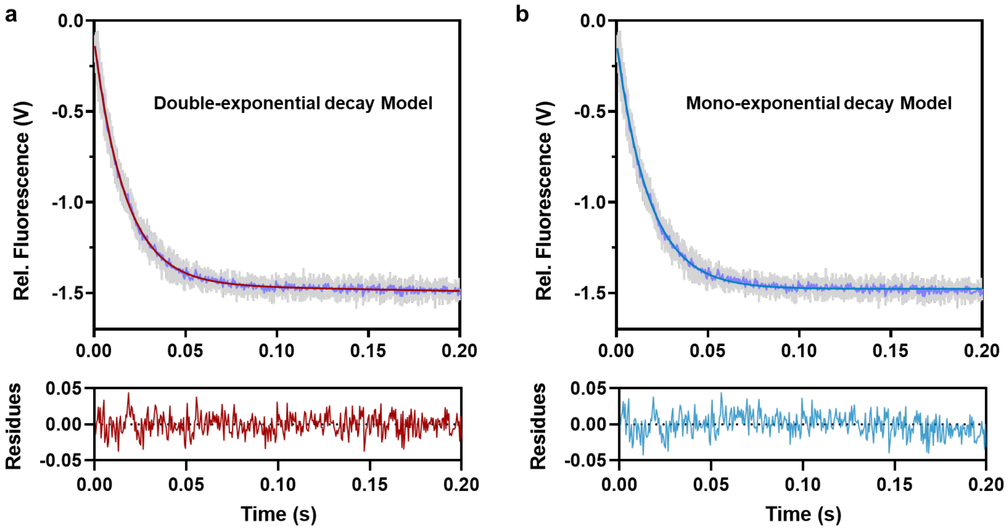

Supplementary Fig 19: Time course of representative binding of NADPH (25 µM) to AKRtyl-W331A (0.2 µM), indicating one step binding compare to WT.

In the time course curve for each concentration, the purple line represents the average of three consecutive trials and the gray area represents the SD (n = 3 independent experiments). **a**, Fitting with the double-exponential decay equation. **b**, Fitting with the mono-exponential decay equation.

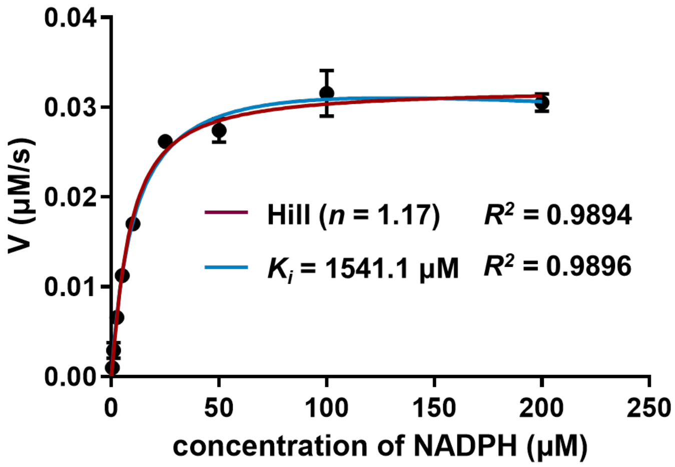

Supplementary Fig 20: Kinetics of AKRtyl-W331A to NADPH.

The data were fitted with the substrate-inhibition equation (blue line; R^2 = 0.9896) and the Hill equation (red line; R^2 = 0.9894). Error bars indicate means ± SD (n = 3 independent experiments).

a **The conformational selection model:**

$$k_{obs} = \left[k_{-r} + k_r + k_{off} + k_{on}[L] - \sqrt{\left(k_{off} + k_{on}[L] - k_{-r} - k_r\right)^2 + 4k_{-r}k_{on}[L]}\right]/2$$

$$K_{d,app} = \frac{k_{off}}{k_{on}}\left(\frac{k_r + k_{-r}}{k_r}\right)$$

The induced fit model:

$$k_{obs} = \left[k_{-r} + k_r + k_{off} + k_{on}[L] - \sqrt{\left(k_{off} + k_{on}[L] - k_{-r} - k_r\right)^2 + 4k_r k_{off}}\right]/2$$

$$K_{d,app} = \frac{k_{off}}{k_{on}}\left(\frac{k_r}{k_r + k_{-r}}\right)$$

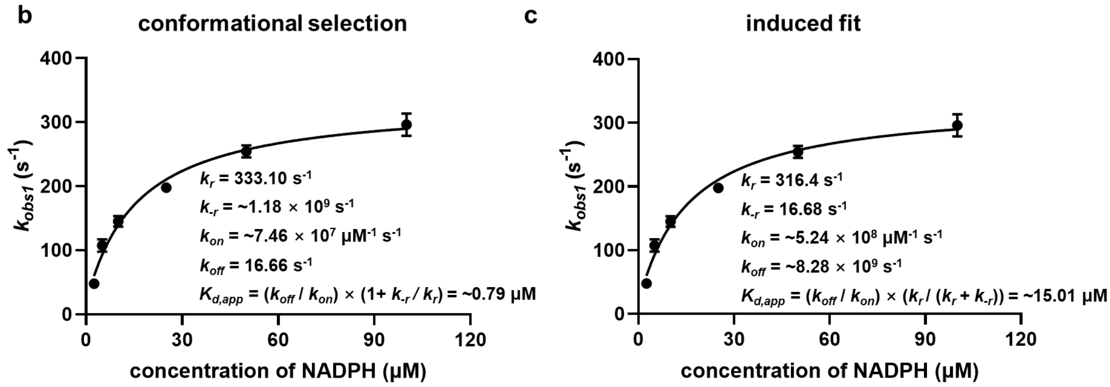

Supplementary Fig 21: Conformational selection (the simplest form of the MWC) model and induced fit (the simplest form of the KNF) model fitting for AKRtyl k_{obs1}-[NADPH] data.

a, The conformational selection and induced fit model. Where k_{obs} is the observed rate constant, [L] is the ligand concentration, k_r and k_{-r} refer to the rate of conformational isomerization and backward, respectively, k_{on} and k_{off} refer to the rate of ligand association and dissociation, respectively, $K_{d,app}$ is the apparent dissociation constant and the parameter accessible to experimental measurements of the system at equilibrium. **b**, Conformational selection model fitting for k_{obs1}-[NADPH] data. **c**, Induced fit model fitting for k_{obs1}-[NADPH] data. Error bars indicate means ± SD (n = 3 independent experiments).

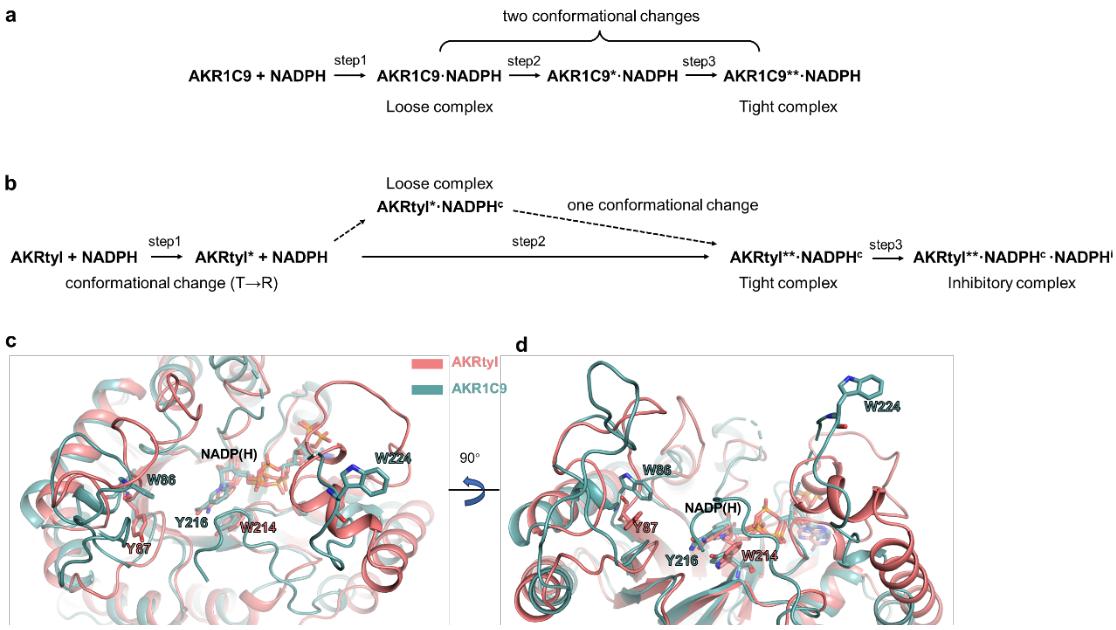

Supplementary Fig 22: Comparison of NADP (H) binding to AKRtyl and AKR1C9.

a-b, Three-step model for NADP(H) binding to AKR1C9 and AKRtyl. **c-d**, Structural comparison of AKRtyl and AKR1C9 binding NADP(H).

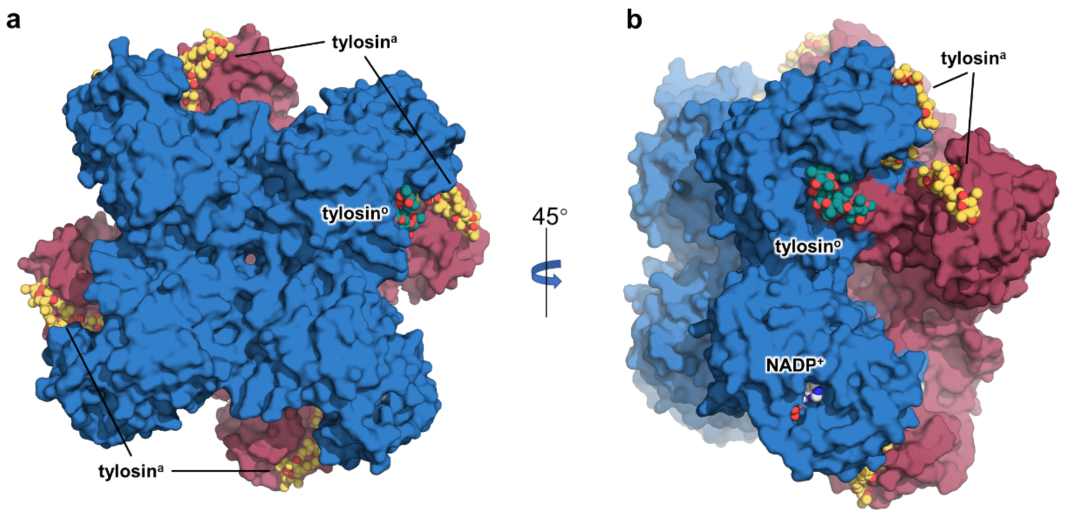

Supplementary Fig 23: Surface representation of AKRtyl octamer bound with two tylosins.

a, **b**, Top and side views (45° rotation) of the AKRtyl octamer binding two tylosins. The tylosin[o] binds at the active site (green spheres) and the tylosin[a] binds at the allosteric site (yellow spheres).

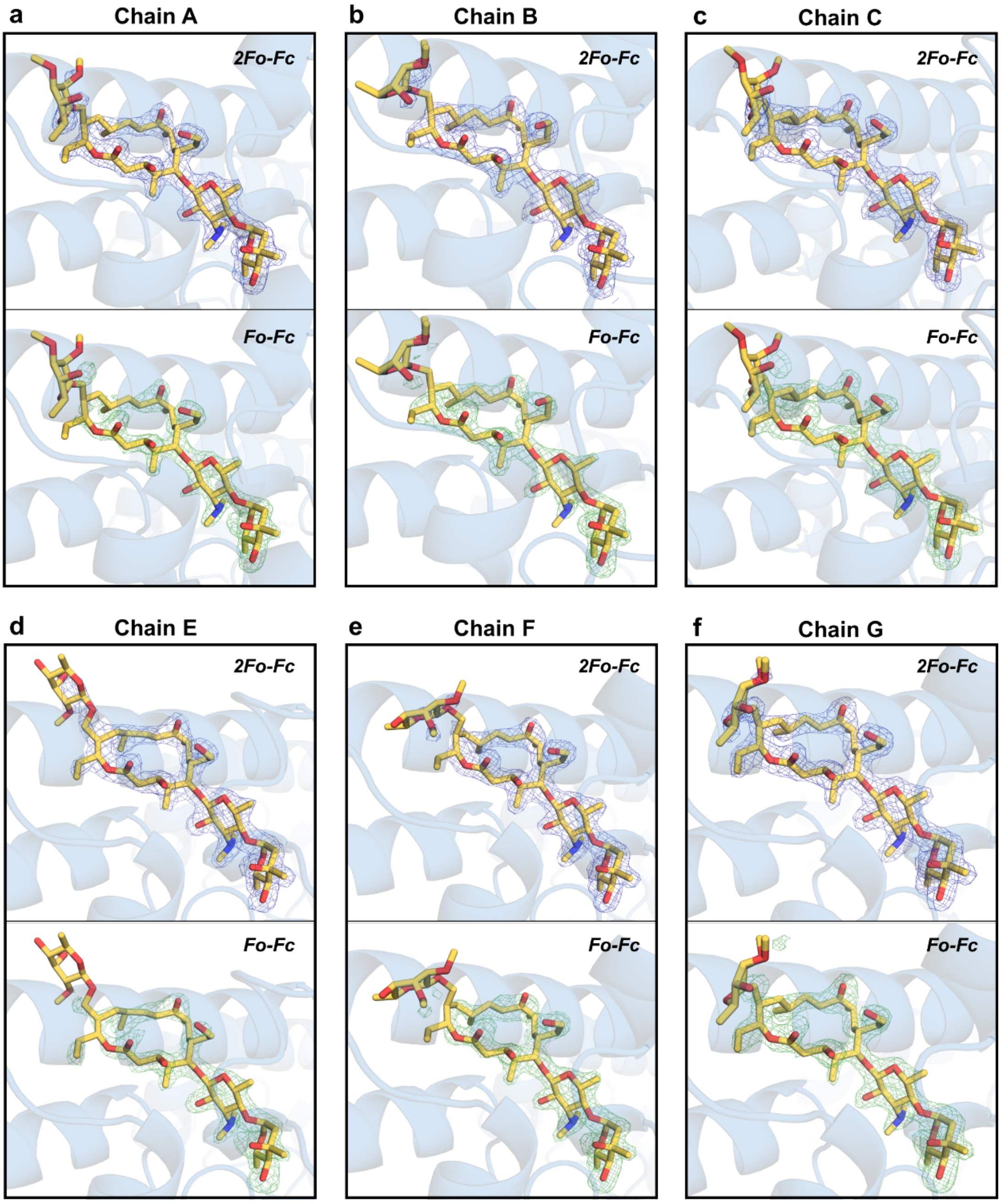

Supplementary Fig 24: Electron density maps for allosteric tylosins.

$2F_o$-F_c electron density maps (shown in blue) for allosteric tylosins in six chains (up) contoured at 1.0σ; F_o-F_c omit maps (shown in green) for allosteric tylosins in six chains (down) contoured at 3.0σ.

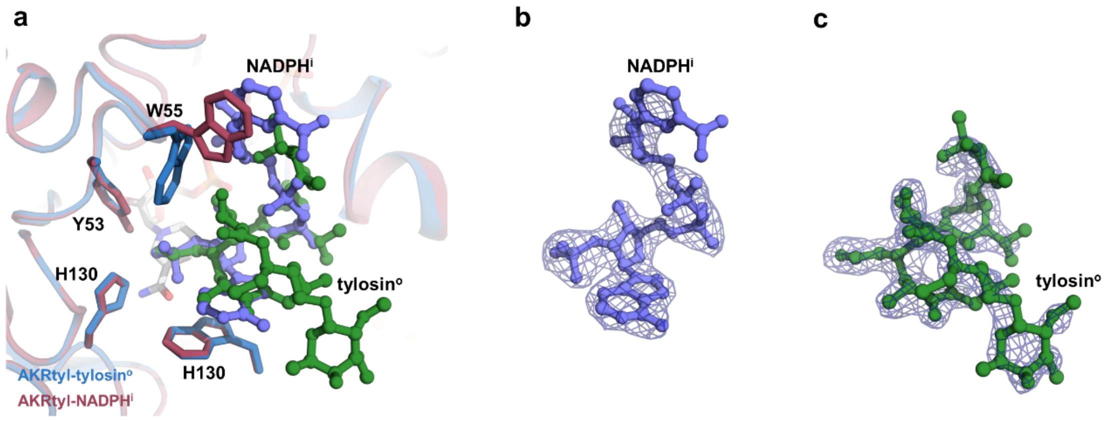

Supplementary Fig 25: Structural comparison of the tylosin^{o} and the NADPH^{i} both bound to the active site.

a, Alignment of the monomer structure of AKRtyl-tylosin^{o} complex (blue) with the monomer structure of AKRtyl-NADPH^{i} complex (red) shows a C_{α}-RMSD of 0.101 Å. The tylosin^{o} and the NADPH^{i} are shown as green and purple ball-and-stick, respectively. The key residues are shown as stick. **b, c**, The NADPH^{i} and the tylosin^{o} structural model are superposed with the corresponding $2F_o$-F_c electron density map contoured at 1.0 σ and 0.7σ. These two molecules have different electron densities.

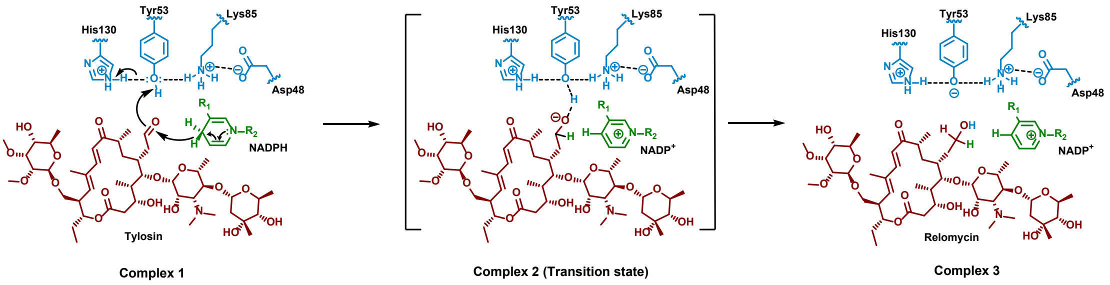

Supplementary Fig 26: Proposed AKRtyl-catalyzed reduction mechanism.

In complex 1, the catalytic residue Tyr53 forms a hydrogen bond with the tylosin carbonyl, resulting in carbonyl polarization and accelerating the hydride transfer of the *pro*-R hydrogen from the nicotinamide ring of NADPH to the carbonyl carbon of the substrate. The hydrogen bond network provided by His130, Lys85, and Asp48 serves to lower the *pKa* of Tyr53, facilitating proton transfer. Complex 2 shows a transition state in which after hydride transfer to the acceptor carbonyl, the carbonyl is protonated by tyrosine via a proton relay that involves tetrad residues to bulk water. The reduced carbonyl then dissociates from the acid-base catalyst and a net charge on the tyrosinated anion is stabilized by the hydrogen bonding network (complex 3).

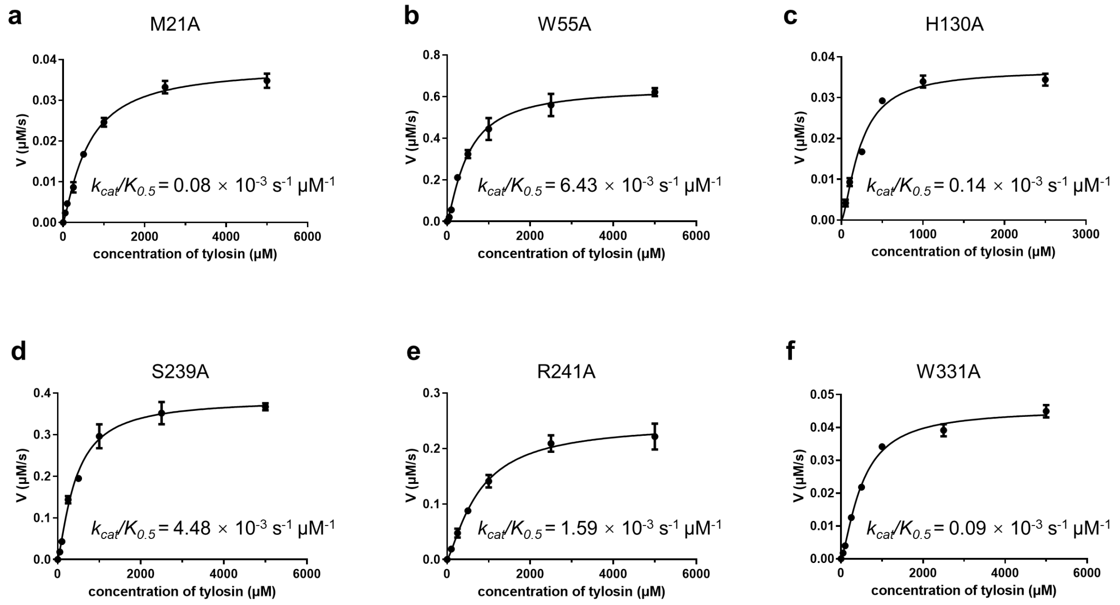

Supplementary Fig 27: Kinetics of AKRtyl variants of the orthosteric site.

a, M21A; **b**, W55A; **c**, H130A; **d**, S239A; **e**, R241A; **f**, W331A.

Error bars indicate means ± SD (n = 3 independent experiments).

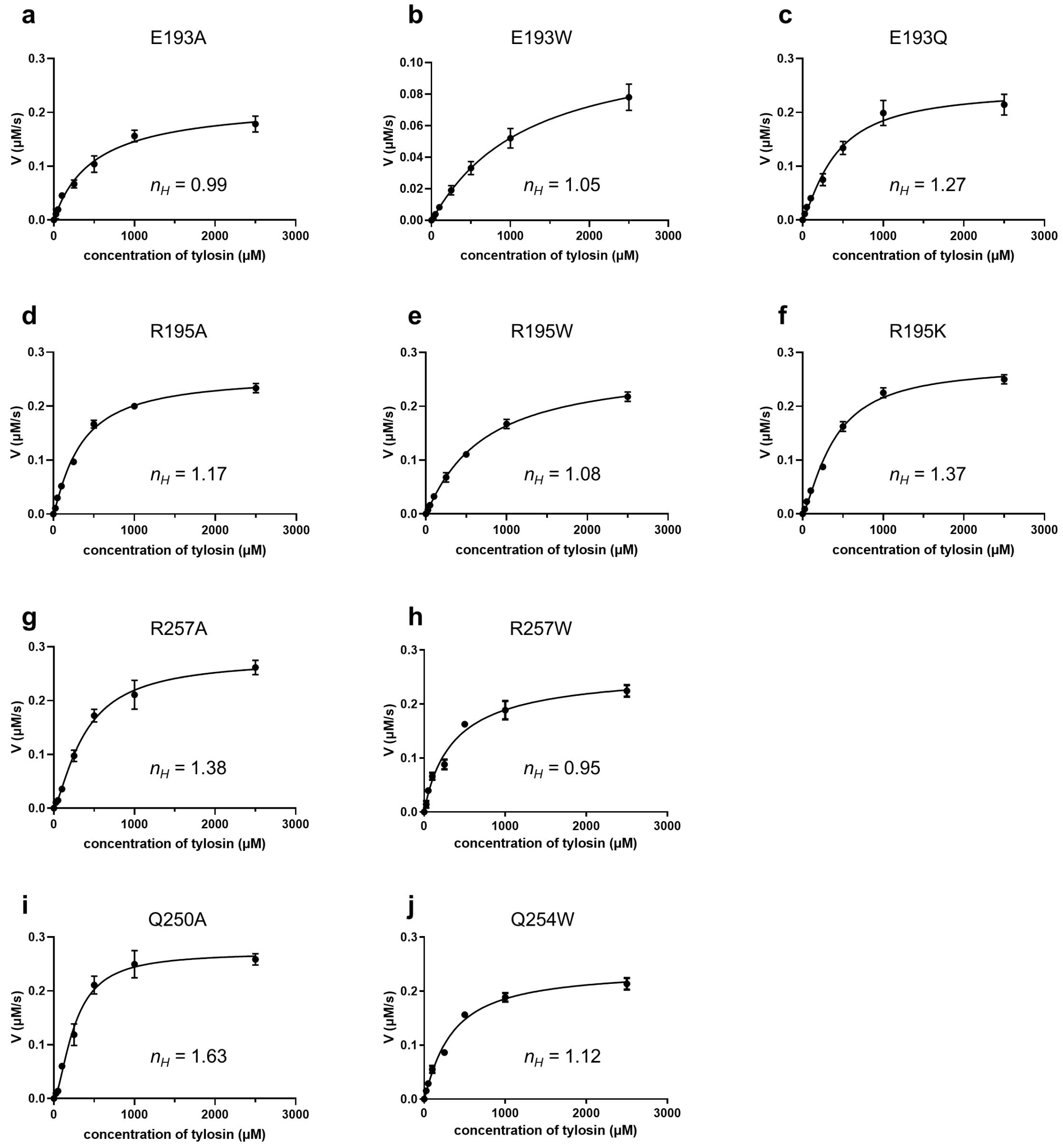

Supplementary Fig 28: Kinetics of AKRtyl variants of the allosteric site.

a, E193A; **b**, E193W; **c**, E193Q; **d**, R195A; **e**, R195W; **f**, R195K; **g**, R257A; **h**, R257W; **i**, Q250A; **j**, Q254W.

Error bars indicate means ± SD (n = 3 independent experiments).

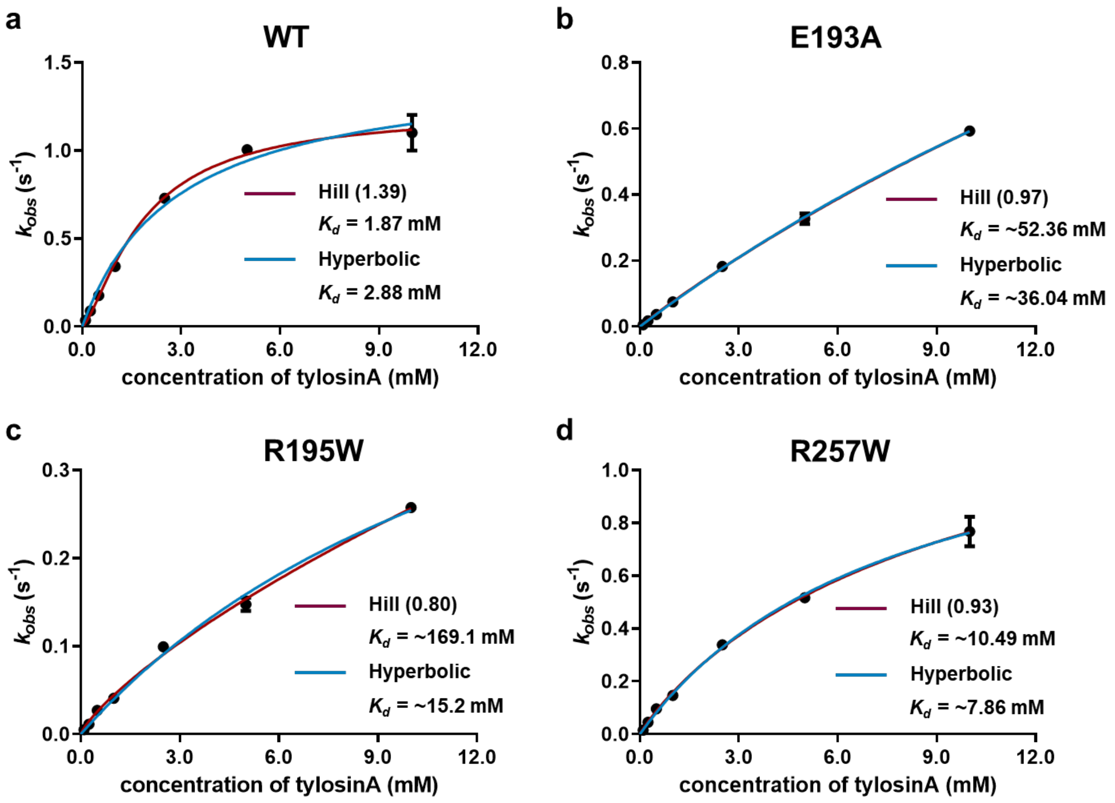

Supplementary Fig 29: The single turnover kinetic curves of AKRtyl and its substrate allosteric binding site mutants to tylosin.

a, WT; **b**, E193A; **c**, R195W; **d**, R257W. The data were fitted with both the hyperbolic equation (blue line) and the allosteric-sigmoidal Hill equation (red line).

Error bars indicate means ± SD (n = 3 independent experiments).

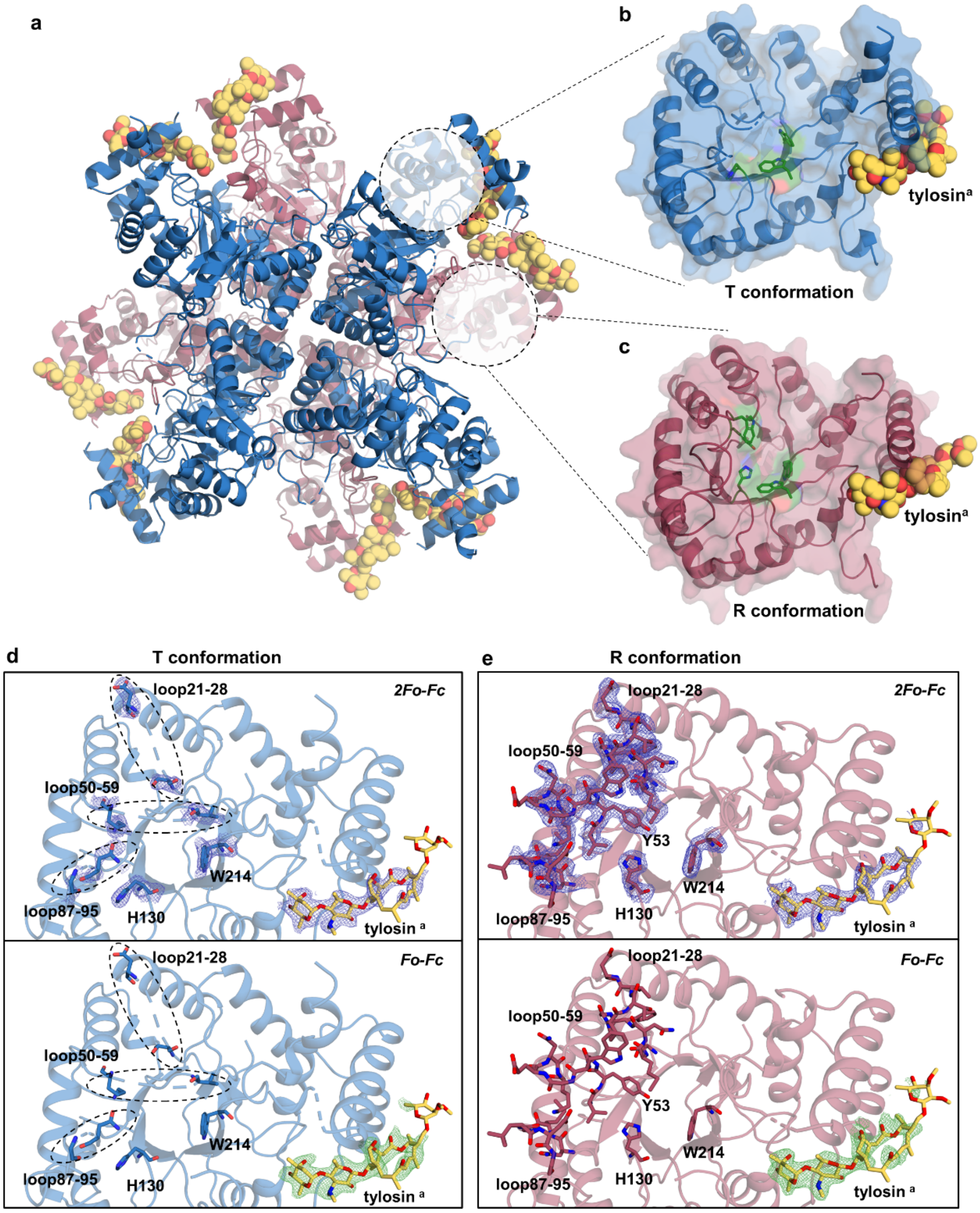

Supplementary Fig 30: Crystal structure of AKRtyl-tyosin complex without NADP(H).

a, The octameric assembly of AKRtyl-tylosin complex which binds 8 tylosins at the allosteric sites. **b**, The dormant conformation with allosteric tylosin. **c**, The active conformation with allosteric tylosin. **d**, 2*Fo-Fc* electron density maps (shown in blue) for allosteric tylosin in T conformation (up) contoured at 1.0σ; *Fo-Fc* omit maps (shown

in green) for allosteric tylosin in T conformation (down) contoured at 2.5σ. **e**, $2F_o$-F_c electron density maps (shown in blue) for allosteric tylosin in R conformation (up) contoured at 1.0σ; F_o-F_c omit maps (shown in green) for allosteric tylosin in R conformation (down) contoured at 2.5σ.

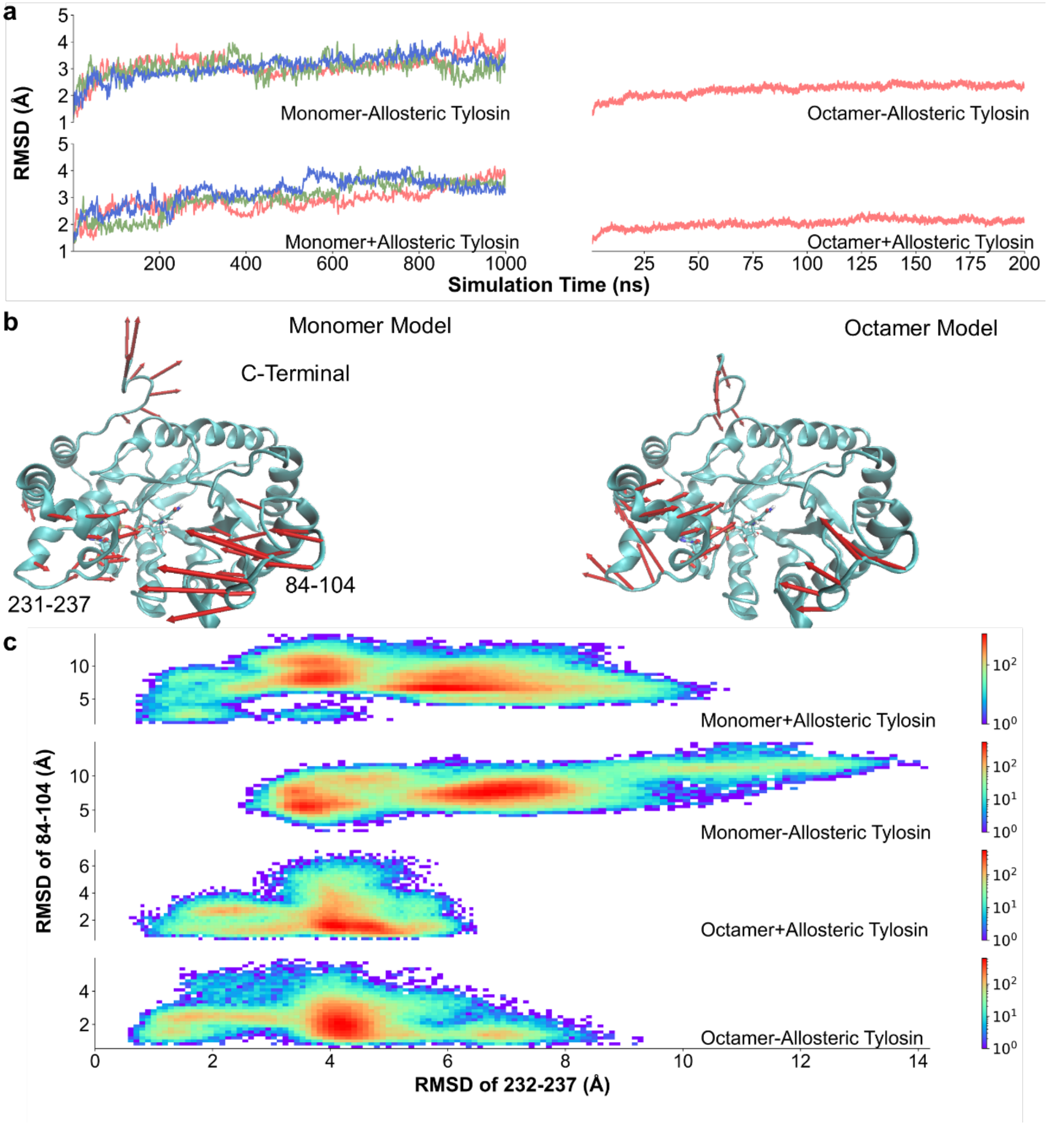

Supplementary Fig 31: Simulation of AKRtyl-NADP(H) complex with/without allosteric tylosin in the absence of orthosteric substrate.

a, The RMSD of the AKRtyl-NADPH monomer (left) during simulation with 3 rounds and octamer (right) with 1 round. **b**, PCA of the trajectories. The swing (red arrows) of two loops (residues 84-104 and 231-237) and the C-terminal accommodate dominant motions in both monomer (left) and octamer (right). **c**, Conformation clustering using the RMSD of loop 232-237 and loop 84-104. The allosterically bound ensemble has additional accessible conformations with further opening of the loop 232-237 in both monomer and octamer.

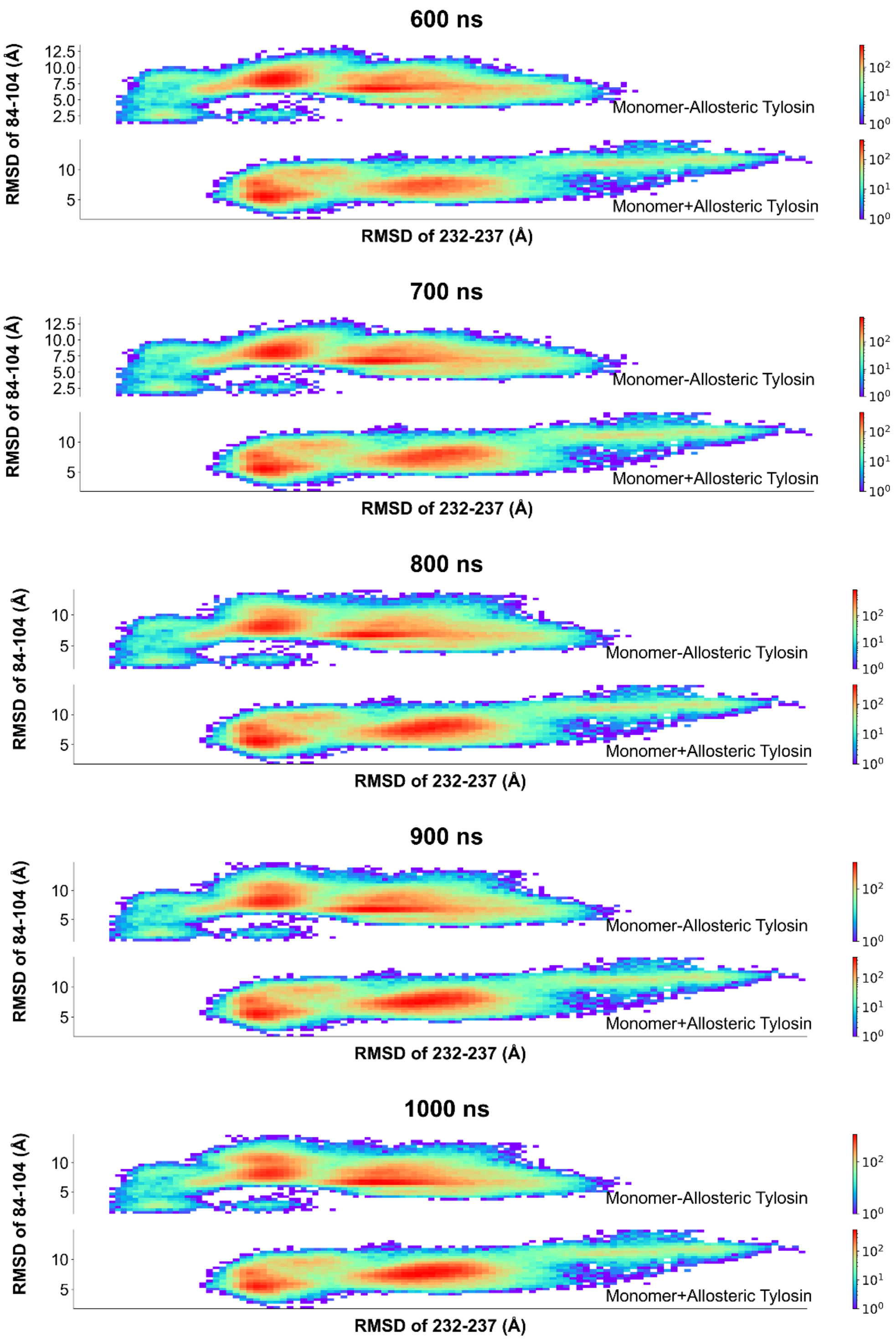

Supplementary Fig 32: Time-independent analysis of conformation populations of monomer models in molecular dynamics.

The populations do not change significantly from 600-1000 ns.

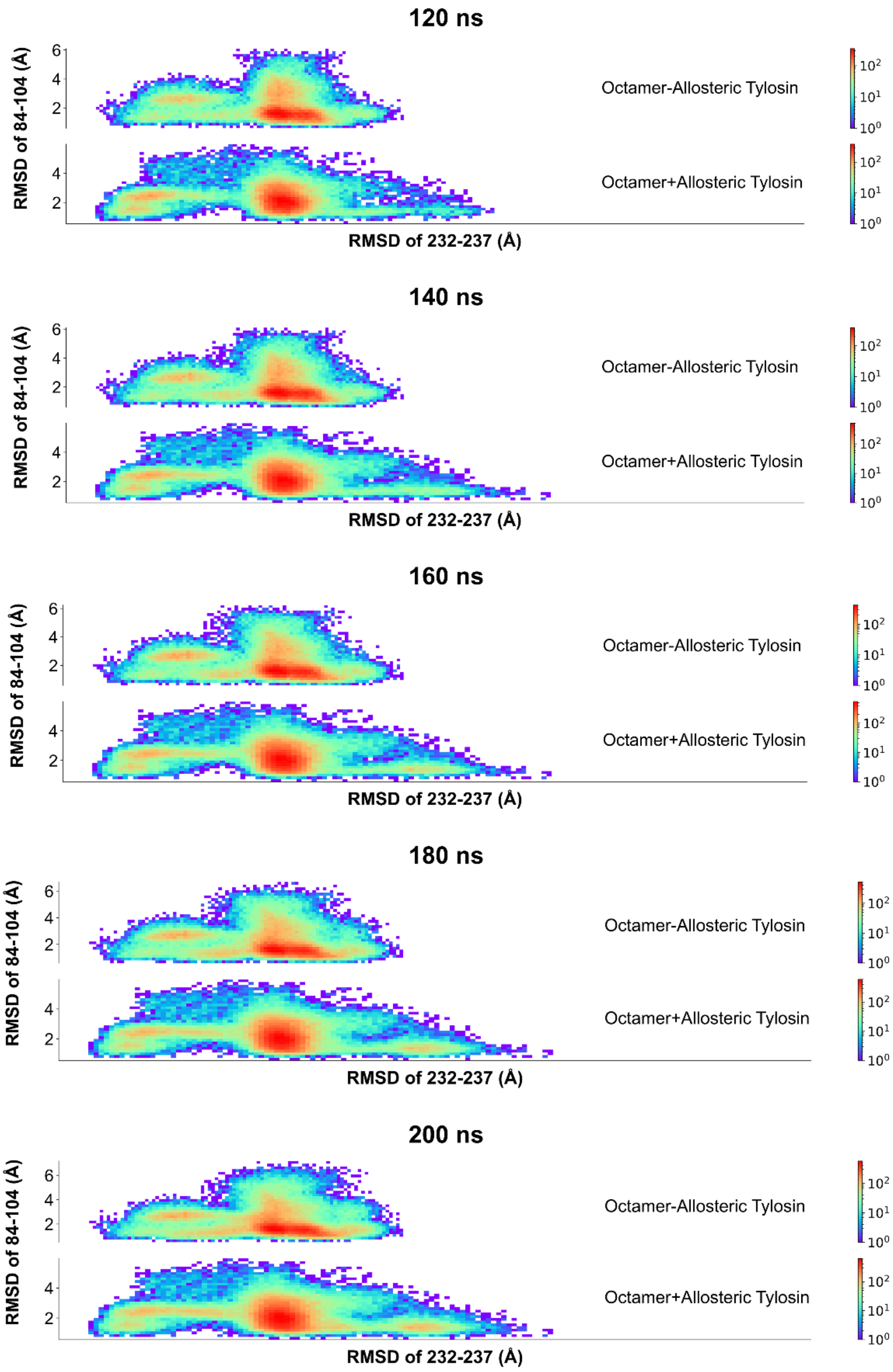

Supplementary Fig 33: Time-independent analysis of conformation populations of octamer models in molecular dynamics.

The populations do not change significantly from 120-200 ns.

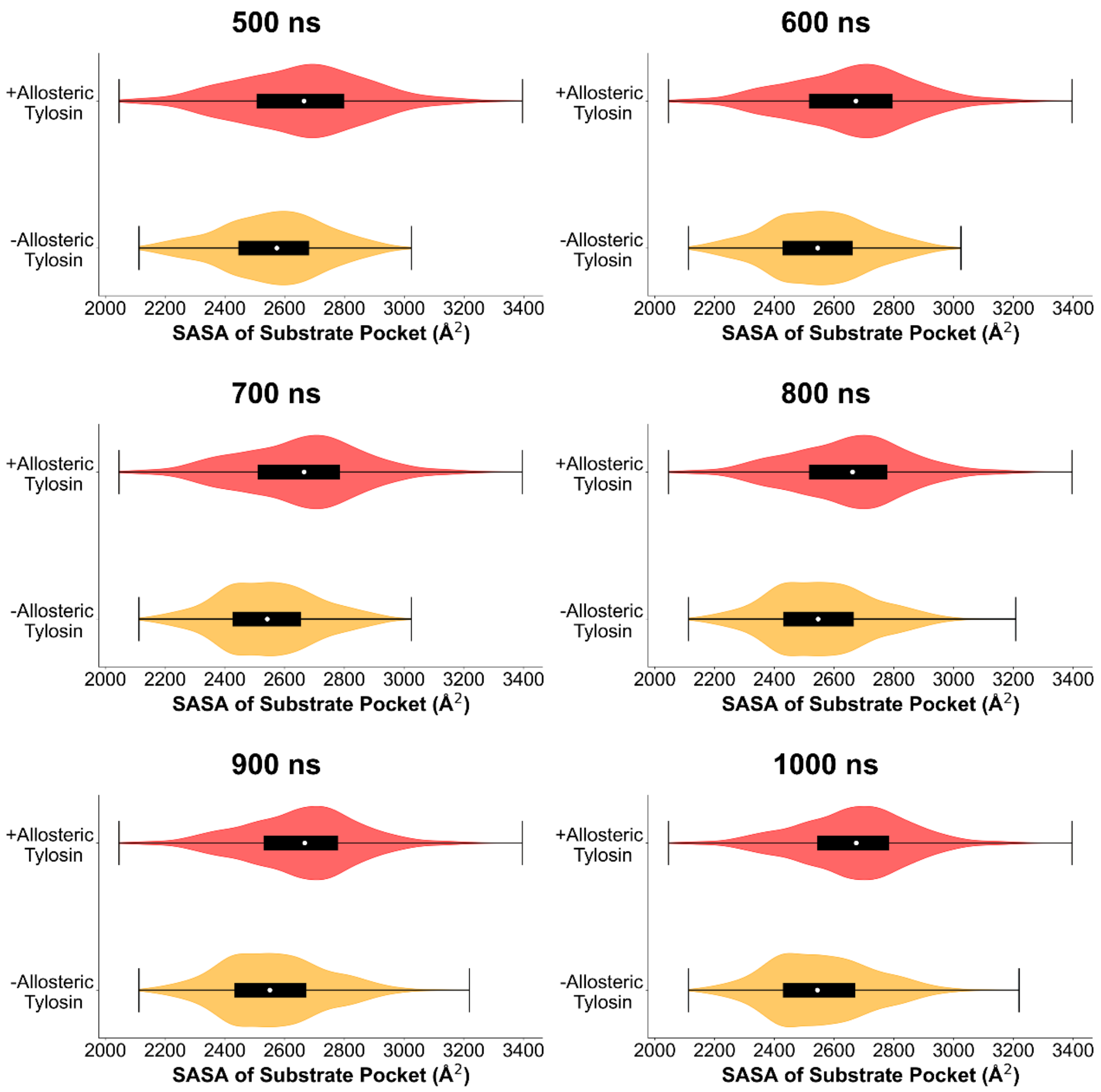

Supplementary Fig 34: Time-independent analysis of volumes of the substrate pockets in monomer models in molecular dynamics.

The volumes do not change significantly from 500-1000 ns. Monomer-Allosteric Tylosin, n=1500; Monomer+Allosteric Tylosin n=758. On violin plots, the upper and lower lines define the range of values while the box defines values between 0.25 and 0.75 quartiles; the white dot points the median.

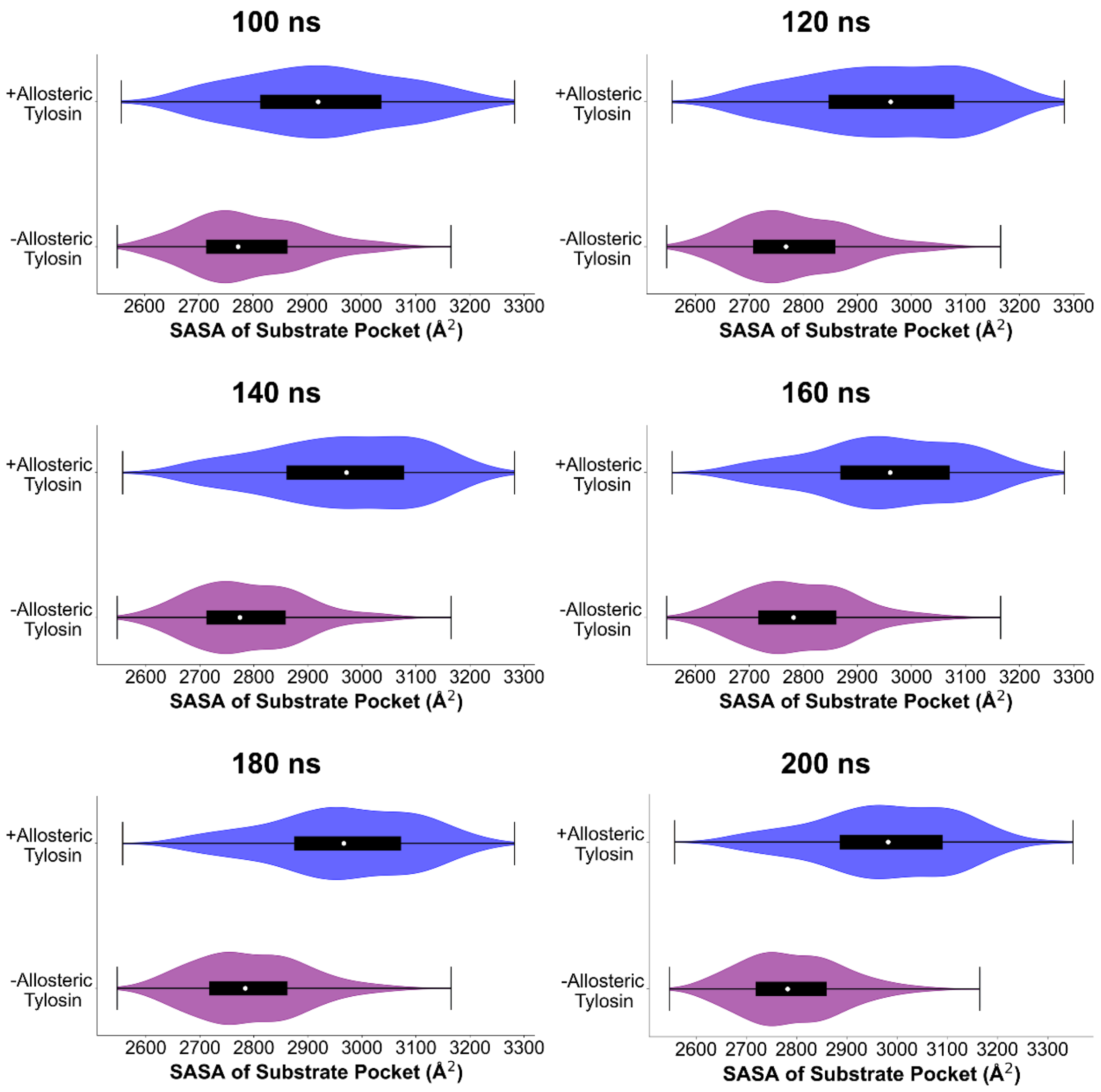

Supplementary Fig 35: Time-independent analysis of volumes of the substrate pockets in octamer models in molecular dynamics.

The volumes do not change significantly from 100-200 ns. Monomer-Allosteric Tylosin, n=1500; Monomer+Allosteric Tylosin n=758. On violin plots, the upper and lower lines define the range of values while the box defines values between 0.25 and 0.75 quartiles; the white dot points the median.

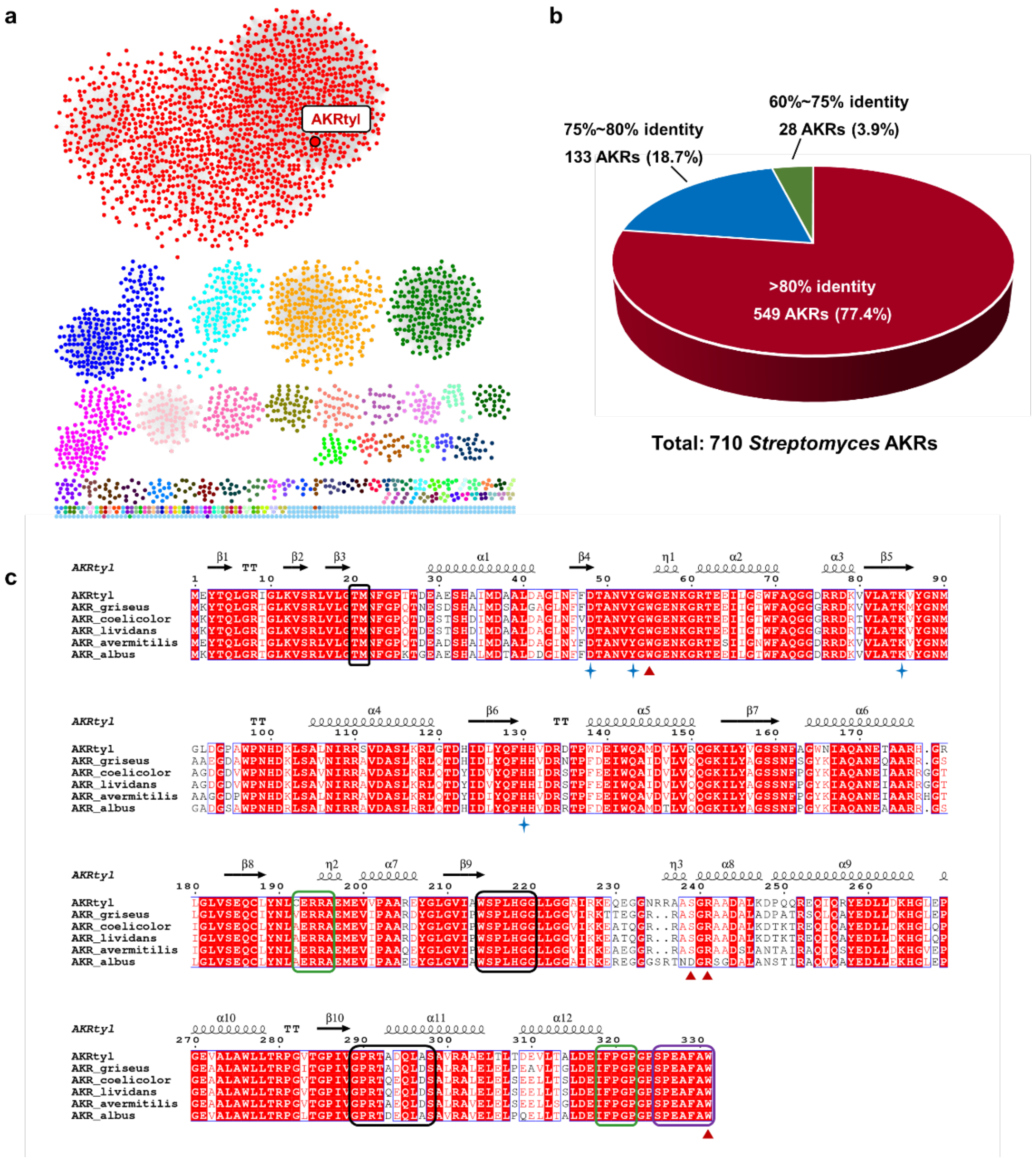

Supplementary Fig 36: The subfamily (AKR12D) represented by AKRtyl is widely distributed in *Streptomyces* with highly conserved features.

a, A complete sequence similarity network (SSN) of AKRs from family 12. The SSN contains 3,898 sequences of AKRs come from family 12. Sequences were compiled with an alignment score of 142 and each node represents a single sequence. AKRtyl is labeled in red and is located in the biggest cluster (subfamily 12D). **b**, The percentage of the sequence identity of AKRtyl and *Streptomyces* AKRs from subfamily 12D. 710 proteins are from *Streptomyces*, accounting for more than half of subfamily 12D, and

over 75% of them have a sequence identity exceeding 80% with AKRtyl. **c**, Multiple sequence alignment of AKRtyl with representative AKRs from *Streptomyces*. Amino acid sequences of AKRs in subfamily 12D from different *Streptomyces* were used for the multiple sequence alignment with AKRtyl. The aligned sequences and their Uniprot accession numbers are: AKR_albus (*S. albus* J1074, A0A6B3HBY1); AKR_coelicolor (*S. coelicolor* M1154, Q9F2Z5); AKR_lividans (*S. lividans* TK24, D6EIK6); AKR_albus (*S. griseus* NBRC 13350, B1VKW1); AKR_avermitilis (*S. avermitilis* ATCC 31267, Q82GK7). Catalytic tetrads are marked with blue asterisks (D48, Y53, K85, H130 in AKRtyl). Substrate binding residues (also NADPH[i] binding residues) are marked with red triangles. The region corresponding to the cofactor NADPH binding site, the allosteric tylosin binding site and the C-terminal tail loop in AKRtyl are boxed in black, green and purple, respectively.

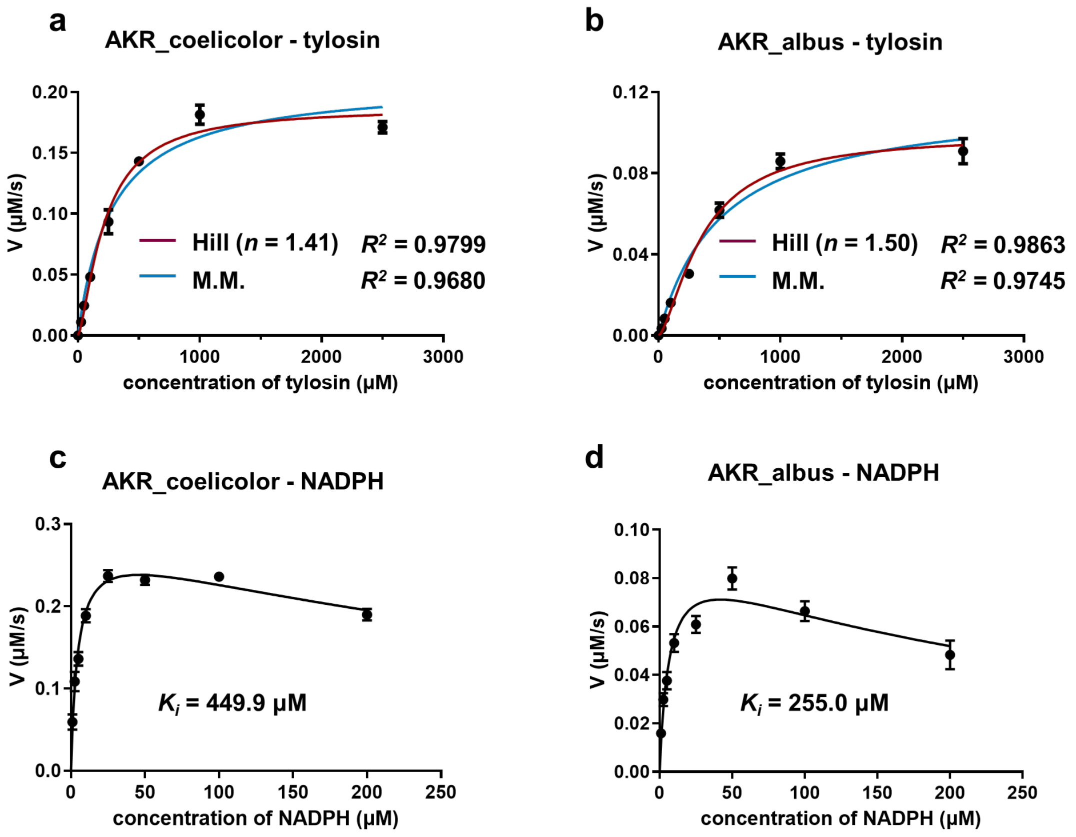

Supplementary Fig 37: Kinetics of AKR_coelicolor and AKR_albus to NADPH and tylosin.

a, Kinetic curves of AKR_coelicolor to tylosin. **b**, Kinetic curves of AKR_albus to tylosin. **c**, Kinetic curves of AKR_coelicolor to NADPH. **d**, Kinetic curves of AKR_albus to NADPH. Error bars indicate means ± SD (n = 3 independent experiments). AKR_coelicolor and AKR_albus both show positive cooperativity with tylosin and inhibited by high concentration NADPH.

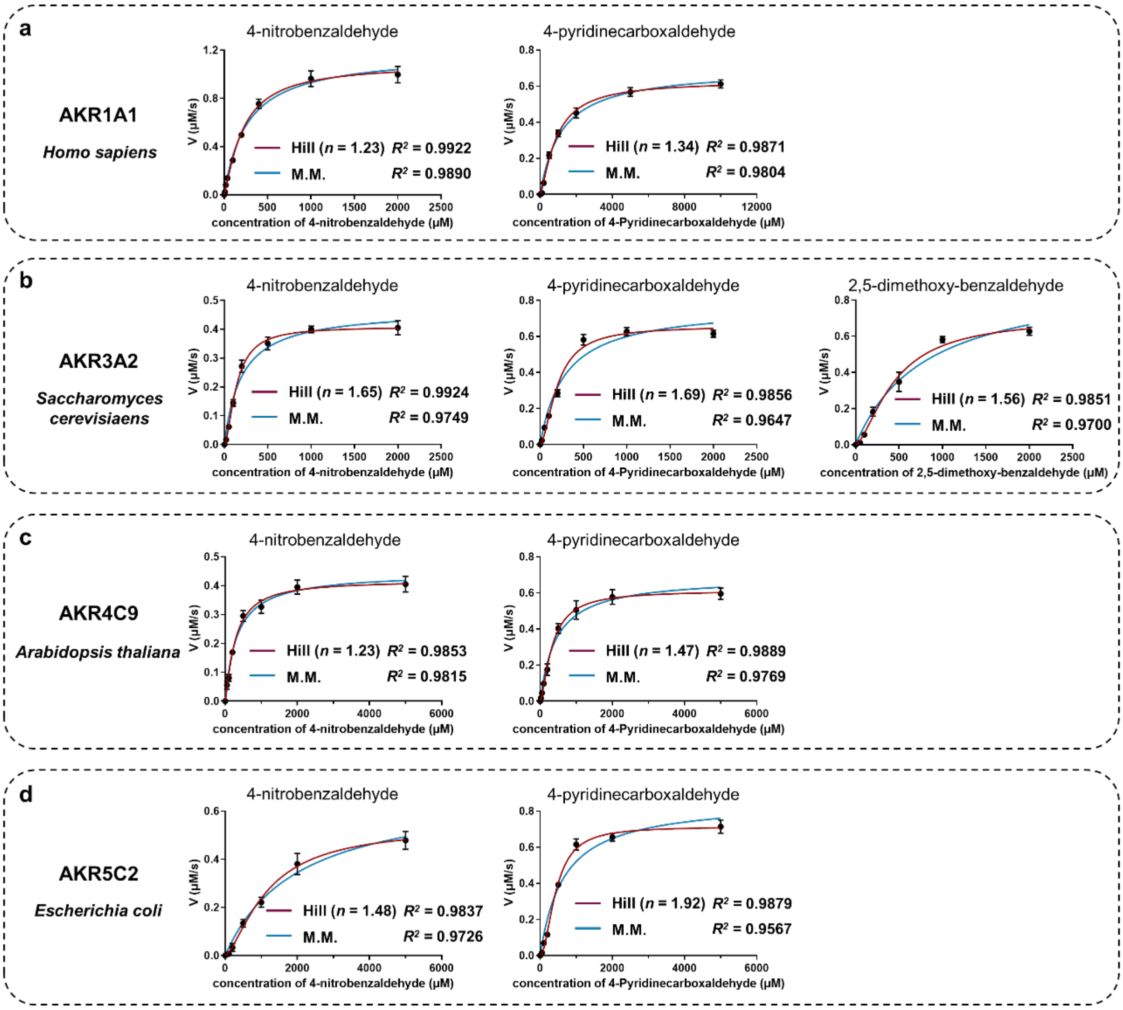

Supplementary Fig 38: Kinetics of other AKRs from three kingdoms of life.

a, Kinetic curves of AKR1A1 from *Homo sapiens* to 4-nitrobenzaldehyde and 4-pyridinecarboxaldehyde. **b**, Kinetic curves of AKR3A2 from *Saccharomyces cerevisiaens* to 4-nitrobenzaldehyde, 4-pyridinecarboxaldehyde and 2,5-dimethoxy-benzaldehyde. **c**, Kinetic curves of AKR4C9 from *Arabidopsis thaliana* to 4-nitrobenzaldehyde and 4-pyridinecarboxaldehyde. **d**, Kinetic curves of AKR5C2 from *Escherichia coli* to 4-nitrobenzaldehyde and 4-pyridinecarboxaldehyde. Error bars indicate means ± SD (n = 3 independent experiments). All four AKRs exhibit varying degrees of positive cooperativity to substrates.

Supplementary Tables

Supplementary Table 1: Activity of AKRtyl on various aldehyde and ketone substrates.

	Substrates	Activity (μM/s/mg protein)
Macrolides	tylosin	265.61 ± 13.45
	desmycosin	163.65 ± 3.63
Aromatic aldehydes	benzaldehyde	104.70 ± 8.54
	Anisaldehyde	82.42 ± 5.40
	4-methoxybenzaldehyde	78.99 ± 5.48
	4-carboxybenzaldehyde	88.63 ± 4.09
	4-nitrobenzaldehyde	572.37 ± 11.96
	4-chlorobenzaldehyde	215.61 ± 6.76
	4-bromobenzaldehyde	233.91 ± 7.47
	4-hydroxybenzaldehyde	23.68 ± 3.03
	o-vanillin	164.28 ± 30.51
	2,5-dimethoxy-benzaldehyde	759.14 ± 23.02
	3,5-dimethylbenzaldehyde	745.36 ± 68.29
	phenylacetaldehyde	78.07 ± 5.37
Aliphatic aldehydes	acetaldehyde	2.11 ± 0.28
	n-butanal	8.37 ± 1.56
	n-hexanal	206.23 ± 16.62
	capraldehyde	141.47 ± 12.42
Heterocyclic aldehydes	4-pyridinecarboxaldehyde	528.07 ± 11.36
	furfural	22.37 ± 3.00
Intracellular useful aldehydes	glyceraldehyde 3-phosphate	15.97 ± 1.37
	erythrose 4-phosphate	56.16 ± 4.68
Ketones	4-hydroxyphenylacetone	N.R. [a]
	4methoxyphenylacetone	N.R. [a]
Carbohydrates	D-glucose	N.R. [a]
	L-arabinose	N.R. [a]

All measurements were carried out in triplicate and are shown as means ± SD (n=3 independent experiments).

[a] N.R. : No reaction

Supplementary Table 2: Comprehensive kinetic data of AKRtyl and its mutants to tylosin in this research. [a]

Proteins		$K_{0.5}$ (μM)	k_{cat} (s^{-1})	$k_{cat}/K_{0.5}$ (s^{-1} μM^{-1})	Hill coeff n [b]
WT		214.51 ± 17.17	1.44 ± 0.05	6.71 × 10^{-3}	1.76 ± 0.19 (Pos. coop.)
WT [c]		278.00 ± 12.32	4.65 ± 0.09	16.72 × 10^{-3}	1.76 ± 0.11 (Pos. coop.)
Allosteric Site Mutants	E193A	501.70 ± 176.30	1.10 ± 0.15	2.20 × 10^{-3}	0.99 ± 0.17 (Loss of coop.)
	E193W	1147.98 ± 334.21	0.56 ± 0.07	0.49 × 10^{-3}	1.05 ± 0.12 (Loss of coop.)
	E193Q	407.32 ± 89.71	1.22 ± 0.12	3.00 × 10^{-3}	1.27 ± 0.23 (Reduced coop.)
	R195A	333.70 ± 32.77	1.28 ± 0.05	3.84 × 10^{-3}	1.17 ± 0.09 (Reduced coop.)
	R195W	690.83 ± 112.26	1.37 ± 0.10	1.98 × 10^{-3}	1.08 ± 0.09 (Loss of coop.)
	R195K	386.60 ± 34.96	1.38 ± 0.06	3.57 × 10^{-3}	1.37 ± 0.12 (Reduced coop.)
	Q250A	257.22 ± 31.58	1.36 ± 0.07	5.29 × 10^{-3}	1.63 ± 0.25 (Pos. coop.)
	Q254W	329.70 ± 46.26	1.20 ± 0.65	3.63 × 10^{-3}	1.12 ± 0.11 (Reduced coop.)
	R257A	382.19 ± 57.17	1.39 ± 0.09	3.64 × 10^{-3}	1.38 ± 0.20 (Reduced coop.)
	R257W	372.01 ± 99.22	1.31 ± 0.13	3.52 × 10^{-3}	0.95 ± 0.13 (Loss of coop.)
Orthosteric Site Mutants	M21A	612.21 ± 72.55 (2.9*fold*↑)	0.048 ± 0.002 (30*fold*↓)	0.08 × 10^{-3} (84*fold*↓)	1.22 ± 0.13
	Y53F			N.R. [d]	
	W55A	499.53 ± 68.84 (2.3*fold*↑)	3.21 ± 0.17 (2.3*fold*↑)	6.43 × 10^{-3} (no change)	1.27 ± 0.17
	H130A	234.80 ± 24.91	0.033 ± 0.002 (50*fold*↓)	0.14 × 10^{-3} (50*fold*↓)	1.46 ± 0.17
	S239A	428.47 ± 50.00 (2.0*fold*↑)	1.92 ± 0.09	4.48 × 10^{-3} (1.5*fold*↓)	1.31 ± 0.16
	R241A	765.08 ± 147.16 (3.5*fold*↑)	1.22 ± 0.10	1.59 × 10^{-3} (4.2*fold*↓)	1.31 ± 0.23
	W331A	509.53 ± 35.88 (2.3*fold*↑)	0.09 ± 0.01 (16*fold*↓)	0.18 × 10^{-3} (40*fold*↓)	1.40 ± 0.11

All measurements were carried out in triplicate and are shown as means ± SD (n=3 independent experiments).

[a] The saturation concentration of NADPH for tylosin kinetic measurements: 200 μM.

[b] The Hill coefficient n of substrate binding as a measure of cooperativity ($n > 1$, positive cooperativity; $n = 1$, no cooperativity; $n < 1$, negative cooperativity).

[c] The saturation concentration of NADPH in this tylosin kinetic assays: 25 μM.

[d] N.R. : No reaction

Supplementary Table 3: Kinetic properties of His-tagged and non-His-tagged enzyme forms of AKRtyl WT and its mutants.

Protein	$K_{0.5}$ (μM)	k_{cat} (s^{-1})	k_{cat}/K_m (s^{-1} μM^{-1})	Hill coeff n [a]	
WT	256.00 ± 20.59	2.08 ± 0.08	8.13×10^{-3}	1.91 ± 0.24	Pos. coop.
WT-cut His_6-tag	264.10 ± 19.60	1.90 ± 0.07	7.19×10^{-3}	1.97 ± 0.24	Pos. coop.
E193A	447.50 ± 165.60	1.21 ± 0.16	2.70×10^{-3}	0.92 ± 0.16	Loss of coop.
E193A-cut His_6-tag	548.20 ± 111.80	1.21 ± 0.10	2.21×10^{-3}	1.03 ± 0.11	Loss of coop.
R257W	407.00 ± 96.85	1.82 ± 0.18	4.47×10^{-3}	1.15 ± 0.19	Reduced coop.
R257W-cut His_6-tag	488.70 ± 73.23	2.12 ± 0.13	4.34×10^{-3}	1.11 ± 0.10	Reduced coop.

All measurements were carried out in triplicate and are shown as means ± SD (n=3 independent experiments).

[a] The Hill coefficient n of substrate binding as a measure of cooperativity ($n > 1$, positive cooperativity; $n = 1$, no cooperativity; $n < 1$, negative cooperativity).

Supplementary Table 4: Summary of kinetic properties of AKRs to various ligands in this research.

AKRs	Ligands [a]	K_m or $K_{0.5}$ (μM)	k_{cat} (s^{-1})	k_{cat}/K_m (s^{-1} μM^{-1})	K_i (μM) [b]	Hill coeff n [c]	
AKRtyl	desmycosin	346.08 ± 22.91	1.14 ± 0.03	3.29×10^{-3}	-	1.48 ± 0.09	Pos. coop.
	4-pyridinecarboxaldehyde	350.64 ± 20.23	6.47 ± 0.39	18.5×10^{-3}	-	1.41 ± 0.08	Pos. coop.
	4-nitrobenzaldehyde	81.76 ± 7.54	3.52 ± 0.13	43.0×10^{-3}	-	1.54 ± 0.18	Pos. coop.
	4-chlorobenzaldehyde	4031.02 ± 450.22	3.34 ± 0.17	0.83×10^{-3}	-	1.41 ± 0.14	Pos. coop.
	n-hexanal	752.41 ± 31.88	1.26 ± 0.03	1.67×10^{-3}	-	2.11 ± 0.17	Pos. coop.
	n-butanal	10720.03 ± 1080.02	1.29 ± 0.05	0.12×10^{-3}	-	1.31 ± 0.12	Pos. coop.
AKR_ coelicolor	tylosin	220.67 ± 21.71	0.94 ± 0.04	4.26×10^{-3}	-	1.41 ± 0.14	Pos. coop.
	NADPH	4.65 ± 0.61	1.43 ± 0.07	307.5×10^{-3}	449.9 ± 107.1	-	Inhibition
AKR_albus	tylosin	363.54 ± 30.13	0.50 ± 0.02	1.37×10^{-3}	-	1.50 ± 0.14	Pos. coop.
	NADPH	6.83 ± 1.71	0.47 ± 0.05	68.8×10^{-3}	255.0 ± 91.4	-	Inhibition
AKR1A1 (*H. sapiens*)	4-pyridinecarboxaldehyde	900.18 ± 74.09	3.14 ± 0.10	3.49×10^{-3}	-	1.34 ± 0.11	Pos. coop.
	4-nitrobenzaldehyde	216.62 ± 15.88	5.43 ± 0.15	25.1×10^{-3}	-	1.23 ± 0.08	Pos. coop.
AKR3A2 (*S. cerevisiaens*)	4-pyridinecarboxaldehyde	201.90 ± 14.73	3.27 ± 0.10	16.2×10^{-3}	-	1.69 ± 0.16	Pos. coop.
	4-nitrobenzaldehyde	140.45 ± 6.84	2.04 ± 0.04	14.5×10^{-3}	-	1.65 ± 0.11	Pos. coop.
	2,5-dimethoxy-benzaldehyde	449.39 ± 48.42	3.53 ± 0.19	7.85×10^{-3}	-	1.56 ± 0.16	Pos. coop.
AKR4C9 (*A. thaliana*)	4-pyridinecarboxaldehyde	337.83 ± 23.15	3.06 ± 0.08	9.06×10^{-3}	-	1.47 ± 0.11	Pos. coop.
	4-nitrobenzaldehyde	276.67 ± 24.82	2.09 ± 0.06	7.55×10^{-3}	-	1.23 ± 0.10	Pos. coop.
AKR5C2 (*E. coli*)	4-pyridinecarboxaldehyde	440.01 ± 25.52	3.57 ± 0.09	8.11×10^{-3}	-	1.92 ± 0.17	Pos. coop.
	4-nitrobenzaldehyde	1165.96 ± 134.92	2.69 ± 0.17	2.31×10^{-3}	-	1.48 ± 0.17	Pos. coop.

All measurements were carried out in triplicate and are shown as means ± SD (n=3 independent experiments).

[a] The saturation concentration of NADPH for substrate kinetic measurements: 200 μM; the saturation concentration of tylosin for NADPH kinetic measurements: 5 mM;

[b] The inhibition constant.

[c] The Hill coefficient n of substrate binding as a measure of cooperativity ($n > 1$, positive cooperativity; $n = 1$, no cooperativity; $n < 1$, negative cooperativity).

Supplementary Table 5: Equilibrium constants at different NADPH and tylosin concentrations.

NADPH (μM) [a]	5	10	25	50	100	200
Keq	0.86 ± 0.04	20.76 ± 8.73	44.02 ± 5.88	81.37 ± 2.38	10.50 ± 1.56	0.89 ± 0.03
**Tylosin (μM) [b]**	**25**	**50**	**100**	**250**	**500**	**1000**
Keq	2.18 ± 1.31	11.70 ± 3.46	47.26 ± 9.14	33.77 ± 2.13	51.45 ± 12.10	25.16 ± 1.48

All measurements were carried out in triplicate and are shown as means ± SD (n=3 independent experiments).

[a] The concentration of tylosin in the reaction system was fixed at 50 μM.

[b] The concentration of NADPH in the reaction system was fixed at 100 μM.

Supplementary Table 6: X-ray crystallographic data collection and refinement statistics.

	AKRtyl apo1	AKRtyl apo2	1st Purified AKRtyl	2nd Purified AKRtyl
**PDB ID**	8XR2	8XR3	8JWL	8JWK
**Description**	i. Two octamers without any cofactors<br>ii. Two dormant tetramers<br>iii. Two active tetramers	i. Two active subunits without any cofactors	i. One octamer<br>ii. Two active tetramers each binding 1 NADP(H)	i. Two octamers<br>ii. Two dormant tetramers<br>iii. Two active tetramers, one binding 2 NADPHs and one binding 4 NADP(H)s
**Data Collection**				
Wavelength (Å)	0.97861	0.97861	0.97915	0.97915
Space group	$P\,4_3$	I 4	$P\,2_1\,2_1\,2$	$P\,4_3$
Cell dimensions				
a, b, c (Å)	110.8, 110.78, 561.12	109.33, 109.33, 140.99	167.97, 183.09, 102.34	111.89, 111.89, 563.24
α, β, γ (°)	90.00, 90.00, 90.00	90.00, 90.00, 90.00	90.00, 90.00, 90.00	90.00, 90.00, 90.00
Resolution (Å)	50.00-2.39 (2.48-2.39)	50.00-2.31 (2.39-2.31)	50.00-2.30 (2.34-2.30)	50.00-2.32 (2.36-2.32)
R_{merge}	0.192 (0.711)	0.224 (1.140)	0.131 (1.064)	0.160 (0.665)
$I/\sigma I$	9.60 (4.10)	10.20 (2.50)	16.80 (2.65)	11.90 (2.00)
$CC_{1/2}$ (%)	99.2 (83.2)	99.7 (83.3)	98.2 (93.3)	96.5 (76.8)
Completeness (%)	100.0 (100.0)	100.0 (100.0)	99.7 (99.9)	100.0 (100.0)
Redundancy	12.7 (13.3)	13.3 (13.7)	10.9 (10.0)	5.6 (5.6)
**Refinement**				
Resolution (Å)	49.67-2.39	34.57-2.31	38.17-2.30	35.38-2.32
No. reflections	264740	36234	139524	295666
R_{work}/R_{free}	0.2188/0.2622	0.1756/0.2149	0.1870/0.2313	0.1815/0.2172
No. atoms	39819	5246	21068	40000
Protein	38173	4947	19989	38066
Water	1646	299	983	1638
Ligand/ion	0	0	96	296
B-factors (Å^2)	31.20	37.28	29.39	46.89
Protein	31.17	37.20	29.45	47.01
Water	31.85	38.55	28.14	42.25
Ligand/ion	-	-	28.99	57.00
R.m.s. deviations				
Bond lengths (Å)	0.009	0.011	0.009	0.017
Bond angles (°)	0.988	1.319	1.057	1.422

Values in parentheses are for the highest-resolution shell.

	AKRtyl-NADP(H) (1:1.2)	AKRtyl-NADP(H) (1:10)	AKRtyl-NADP^{+}-tylosin	AKRtyl-tylosin
**PDB ID**	8XR4	8JWN	8JWM	8JWO
**Description**	i. One octamer<br>ii. Two active tetramers<br>iii. Each subunit binds 1 NADP(H)	i. One octamer<br>ii. Two active tetramers<br>iii. Each subunit binds 2 NADP(H)s	i. One octamer<br>ii. Two active tetramers<br>iii. Six subunits bind tylosin[a]<br>iv. Each subunit active site has an incomplete tylosin electron density, with one subunit placed into tylosin[o].	i. Two octamers<br>ii. Two dormant tetramers<br>iii. Two active tetramers<br>iv. Each subunit binds a tylosin[a].
**Data Collection**				
Wavelength (Å)	0.97915	0.97852	0.97852	0.97915
Space group	$P\ 2\ 2_1\ 2_1$	$P\ 2\ 2_1\ 2_1$	$P\ 2\ 2_1\ 2_1$	$P\ 4_3$
Cell dimensions				
a, b, c (Å)	82.88, 197.64, 199.98	83.13, 198.73, 200.47	83.01, 198.31, 199.99	111.02, 111.02, 560.04
α, β, γ (º)	90.00, 90.00, 90.00	90.00, 90.00, 90.00	90.00, 90.00, 90.00	90.00, 90.00, 90.00
Resolution (Å)	82.88-1.94 (2.01-1.94)	50.00-2.26 (2.30-2.26)	50.00-1.93 (1.96-1.93)	50.00-2.25 (2.29-2.25)
R_{merge}	0.177 (1.172)	0.126 (0.985)	0.133 (0.766)	0.169 (0.812)
$I/\sigma I$	9.82 (2.31)	17.14 (2.00)	15.60 (2.33)	11.30 (2.50)
$CC_{1/2}$ (%)	99.8 (87.9)	98.3 (86.4)	95.7 (78.8)	99.1 (69.9)
Completeness (%)	100.0 (99.9)	100.0 (100.0)	99.9 (99.9)	100.0 (100.0)
Redundancy	12.1 (12.8)	12.1 (12.0)	6.2 (6.0)	6.8 (6.9)
**Refinement**				
Resolution (Å)	82.88-2.30	31.71-2.25	35.15-1.93	49.74-2.25
No. reflections	242286	155563	244885	314585
R_{work}/R_{free}	0.1692/0.1999	0.1680/0.2128	0.1642/0.1979	0.1810/0.2165
No. atoms	23216	22285	23779	41183
Protein	20448	20424	20444	38012
Water	2384	1093	2503	2147
Ligand/ion	384	768	832	1024
B-factors (Å^2)	29.33	40.28	21.91	37.37
Protein	28.59	39.72	20.66	36.08
Water	33.87	39.53	28.34	35.64
Ligand/ion	28.07	56.36	33.32	88.78
R.m.s. deviations				
Bond lengths (Å)	0.008	0.009	0.009	0.012
Bond angles (º)	0.0920	1.144	1.111	1.582

Values in parentheses are for the highest-resolution shell.

Supplementary Table 7: Fitting of the fluorescent quenching curves of NADPH to AKRtyl with either double or single exponential model.

NADPH (μM)	Model	$(\Delta F)_1$ (V)	k_{obs1} NADPH[c] Step2 (s^{-1}) [a]	$(\Delta F)_2$ (V)	k_{obs2} 2st NADPH[i] Step3 (s^{-1}) [b]	R^2
100	Double exponential	0.97 ± 0.03	296.10 ± 17.54	0.59 ± 0.03	51.05 ± 2.17	0.9840
	Single exponential	1.23 ± 0.02	99.36 ± 2.08	-	-	0.9587
50	Double exponential	1.42 ± 0.03	254.60 ± 9.58	0.70 ± 0.03	46.20 ± 1.65	0.9919
	Single exponential	1.73 ± 0.02	103.40 ± 1.93	-	-	0.9671
25	Double exponential	1.75 ± 0.04	197.80 ± 6.84	0.84 ± 0.05	47.30 ± 1.82	0.9953
	Single exponential	2.27 ± 0.02	100.1 ± 1.37	-	-	0.9819
10	Double exponential	1.26 ± 0.08	145.20 ± 8.52	1.13 ± 0.08	44.55 ± 1.81	0.9958
	Single exponential	2.16 ± 0.01	70.36 ± 0.73	-	-	0.9898
5	Double exponential	2.10 ± 0.03	70.77 ± 1.31	0.24 ± 0.03	16.12 ± 1.87	0.9956
	Single exponential	2.24 ± 0.01	58.70 ± 0.42	-	-	0.9925
2.5	Double exponential	1.65 ± 0.01	47.94 ± 0.44	0.25 ± 0.12	0.94 ± 0.64	0.9920
	Single exponential	1.66 ± 0.01	42.83 ± 0.33	-	-	0.9856

All measurements were carried out in triplicate and are shown as means ± SD (n=3 independent experiments).

[a] The binding process of the NADPH[c] corresponds to the step2 of the three-step model.

[b] The binding process of the NADPH[i] corresponds to the step3 of the three-step model.

Supplementary Table 8: Fitting of the fluorescent quenching curves of NADPH to AKRtyl-W331A with either double or single exponential model.

NADPH (μM)	Model	(ΔF)$_1$ (V)	k_{obs1} (s^{-1})	(ΔF)$_2$ (V)	k_{obs2} (s^{-1})	R^2	
100	Double exponential	1.555 ± 0.01	102.04 ± 1.45	0.09 ± 0.01	10.95 ± 3.19	0.9914	Amplitude and rate of the 2nd process are weak
	Single exponential	1.58 ± 0.01	91.75 ± 0.76	-	-	0.9933	
50	Double exponential	1.41 ± 0.03	79.33 ± 1.70	0.12 ± 0.03	18.62 ± 4.05	0.9963	
	Single exponential	1.49 ± 0.01	69.56 ± 0.48	-	-	0.9954	
25	Double exponential	1.35 ± 0.01	58.74 ± 0.81	0.38 ± 4.81	0.51 ± 6.94	0.9970	Poor fit
	Single exponential	1.36 ± 0.01	56.08 ± 0.34	-	-	0.9966	
10	Double exponential			unfit			
	Single exponential	0.90 ± 0.01	34.17 ± 0.29	-	-	0.9942	
5	Double exponential			unfit			
	Single exponential	0.57 ± 0.01	27.94 ± 0.36	-	-	0.9875	
2.5	Double exponential			unfit			
	Single exponential	0.33 ± 0.01	23.76 ± 0.53	-	-	0.9677	

All measurements were carried out in triplicate and are shown as means ± SD (n=3 independent experiments).

Supplementary Table 9: Fit of the equilibrium state fluorescent quenching of NADPH to AKRtyl-WT and 331A with three models.

Protein	Model	K_{d1} NADPH[c] Step2 （μM）[a]	n NADPH[c]	K_{d2} NADPH[i] Step3 （μM）[b]	R^2
WT	Two-step Hill	0.45 ± 0.08	1.22 ± 0.19	53.54 ± 13.18	0.9591
	Two-step	0.55 ± 0.08	-	68.16 ± 16.69	0.9587
	One-step	2.24 ± 0.25	-	-	0.8783
W331A	Two-step Hill	4.14 ± 1.84	1.18 ± 0.26	92.75 ± 33.79	0.9766
	Two-step	6.40 ± 1.62	-	132.02 ± 51.14	0.9762
	One-step	19.96 ± 1.39	-	-	0.9639

All measurements were carried out in triplicate and are shown as means ± SD (n=8 independent experiments).

[a] The binding process of the NADPH[c] corresponds to the step2 of the three-step model.

[b] The binding process of the NADPH[i] corresponds to the step3 of the three-step model.

Supplementary Table 10: Setup of Simulation Systems

**Monomer-Allosteric Tylosin**	
PDB and Chain	8JWN:A
Simulation Box Dimensions (Å)	90.5×80.7×91.0
Total Number of Atoms	54235
Total Number of Water Molecules	16365
Counter Ions	15 Na^+
**Monomer+Allosteric Tylosin**	
PDB and Chain	8JWM:B<br>(NADP+ is replaced NADPH in 8JWN:A)
Simulation Box Dimensions (Å)	91.3×90.5×77.1
Total Number of Atoms	52447
Total Number of Water Molecules	15722
Counter Ions	15 Na^+
**Octamer-Allosteric Tylosin**	
PDB and Chain	8JWN:A-H
Simulation Box Dimensions (Å)	121.8×153.0×152.8
Total Number of Atoms	247286
Total Number of Water Molecules	68722
Counter Ions	120 Na^+
**Monomer+Allosteric Tylosin**	
PDB and Chain	"Monomer+Allosteric Tylosin"<br>aligned to each chain of 8JWN
Simulation Box Dimensions (Å)	121.8×153.0×152.8
Total Number of Atoms	247313
Total Number of Water Molecules	68355
Counter Ions	120 Na^+

Supplementary Table 11: Strains and plasmids used in this study.

Strains or plasmids	Relevant properties	Source
**Strains**		
Streptomyces fradiae		
TL-01	Wild tylosin-producing strain	This laboratory
Δ*AKRtyl-gene*	Mutant with AKRtyl deletion in TL-01	This study
Escherichia coli		
DH5α	General cloning and plasmid maintenance	GibcoBRL
ET12567/pUB307	Used for intergeneric conjugation	[6]
BL21(DE3)	F- *ompT hsdSB* (rB- mB-) *gal dcm* (DE3)	Novagen
**Plasmids**		
pYH7	*aac(3)IV*, oriT, P*erm*E*	[7]
pET28a	T7 *lac*, pBR322 origin, neo, His_6-tag	Novagen
pGro7	GroES- GroEL *ori* Cm^r	TAKARA
pYH7-Δ*AKRtyl*	The 2kb upstream gene fragment and 2kb downstream gene fragment of Sp-nadC in TL-01 genome were PCR amplified and insert into *Hind*III site of pYH7 for double crossover to generateΔ*AKRtyl-gene* mutant	This study
pET28a-*AKRtyl*	PCR-amplified *AKRtyl* gene was cloned to the *Nde*I and *Xho*I site of pET28a	This study
pET28a-*AKR1A1*	*AKR1A1* gene was cloned to the *Nde*I and *Xho*I site of pET28a	SYNBIO
pET28a-*AKR3A2*	*AKR3A2* gene was cloned to the *Nde*I and *Xho*I site of pET28a	SYNBIO
pET28a-*AKR4C9*	*AKR4C9* gene was cloned to the *Nde*I and *Xho*I site of pET28a	SYNBIO
pET28a-*AKR5C2*	PCR-amplified *AKR5C2* gene was	This study

	cloned to the *Nde*I and *Xho*I site of pET28a	
pET28a-*AKR_coelicolor*	PCR-amplified *AKR_coelicolor* gene was cloned to the *Nde*I and *Xho*I site of pET28a	This study
pET28a-*AKR_albus*	PCR-amplified *AKR_albus* gene was cloned to the *Nde*I and *Xho*I site of pET28a	This study
pET28a-*AKRtyl*-M21A	Site-directed mutagenesis of methionine 21 to alanine in *AKRtyl*	This study
pET28a-*AKRtyl*-Y53F	Site-directed mutagenesis of tyrosine 53 to phenylalanine in *AKRtyl*	This study
pET28a-*AKRtyl*-W55A	Site-directed mutagenesis of tryptophan 55 to alanine in *AKRtyl*	This study
pET28a-*AKRtyl*-H130A	Site-directed mutagenesis of histidine 130 to alanine in *AKRtyl*	This study
pET28a-*AKRtyl*-E193A	Site-directed mutagenesis of glutamic acid 193 to alanine in *AKRtyl*	This study
pET28a-*AKRtyl*-E193W	Site-directed mutagenesis of glutamic acid 193 to tryptophan in *AKRtyl*	This study
pET28a-*AKRtyl*-E193Q	Site-directed mutagenesis of glutamic acid 193 to glutamine in *AKRtyl*	This study
pET28a-*AKRtyl*-R195A	Site-directed mutagenesis of arginine 195 to alanine in *AKRtyl*	This study
pET28a-*AKRtyl*-R195W	Site-directed mutagenesis of arginine 195 to tryptophan in *AKRtyl*	This study
pET28a-*AKRtyl*-R195K	Site-directed mutagenesis of arginine 195 to lysine in *AKRtyl*	This study
pET28a-*AKRtyl*-S239A	Site-directed mutagenesis of serine 239 to alanine in *AKRtyl*	This study
pET28a-*AKRtyl*-S241A	Site-directed mutagenesis of serine 241 to alanine in *AKRtyl*	This study
pET28a-*AKRtyl*-Q250A	Site-directed mutagenesis of	This study

	glutamine 250 to alanine in *AKRtyl*	
pET28a-*AKRtyl*-Q254W	Site-directed mutagenesis of glutamine 254 to tryptophan in *AKRtyl*	This study
pET28a-*AKRtyl*-R257A	Site-directed mutagenesis of arginine 257 to alanine in *AKRtyl*	This study
pET28a-*AKRtyl*-R257W	Site-directed mutagenesis of arginine 257 to tryptophan in *AKRtyl*	This study
pET28a-*AKRtyl*-W331A	Site-directed mutagenesis of tryptophan 331 to alanine in *AKRtyl*	This study

Supplementary Table 12: Primers used in this study.

Primer	Sequence (5' to 3')	Description
Δ*AKRtyl*-F1	GAGACGGACCACCAGCCAACGCCTTGGAGTGCG	Primers for *AKRtyl* deletion
Δ*AKRtyl*-R1	tcgacctgcaggcatgcAAGCTTCTCCCCGTCCGCGCCCTTCCCG	
Δ*AKRtyl*-F2	TTGGCTGGTGGTCCGTCTCCGGACCGCGCAGGAGC	Primers for *AKRtyl* deletion
Δ*AKRtyl*-R2	agtgcttgcggcagcgtgaagcttGCCCGGCAGGCCTTCGTCGCC	
Δ*AKRtyl-ver*-F	AGCCGCACTACGAGACGGTG	Primers for *AKRtyl* deletion
Δ*AKRtyl-ver*-R	TGCCCGTGCTGCCCAACGTG	
*AKRtyl-Nde*I-F	atcatcatcacagcagcggcctggtgccgcgcggcagccatATGGAATACACGCAACTC	Primers for AKRtyl protein expression
*AKRtyl-Xho*I-F	ttagcagccggatctcagtggtggtggtggtggtgctcgagTCACCAGGCGAATGCCTC	
*AKR5C2-Nde*I-F	tggtgccgcgcggcagccatATGGCTAATCCAACCGTTATTAAG	Primers for AKR5C2 protein expression
*AKR5C2-Xho*I-F	atctcagtggtggtggtggtggtgctcgagTCAGCCGCCGAACTGGTCAG	
*AKR_coelicolor-Nde*I-F	tggtgccgcgcggcagccatatgATGAAGTACACGCAGCTCGG	Primers for AKR_coelicolor protein expression
*AKR_coelicolor-Xho*I-F	agtggtggtggtggtggtgctcgagTCACCATGCGAAGGCTTCGG	
*AKR_albus-Nde*I-F	tggtgccgcgcggcagccatatgATGAAGTACACGCAGCTTGG	Primers for AKR_albus protein expression
*AKR_albus-Xho*I-F	agtggtggtggtggtggtgctcgagTCACCAGGCGAAGGCCTCCG	
AKRtyl-M21A-F	GCCTGGTTCTCGGCACCGCGAACTTCGGCCCCAC	Site-directed mutagenesis of AKRtyl-M21A
AKRtyl-M21A-F	GTGGTGGGGCCGAAGTTCGCGG	

	TGCCGAGAACC	
AKRtyl-Y53F-F	TCGACACCGCCAACGTCTTCGGC TGGGGCGAG	Site-directed mutagenesis of AKRtyl- Y53F
AKRtyl-Y53F-R	GTTCTCGCCCCAGCCGAAGACG TTGGCGGTG	
AKRtyl-W55A-F	ACCGCCAACGTCTACGGCGCGG GCGAGAACAAG	Site-directed mutagenesis of AKRtyl- W55A
AKRtyl-W55A-F	GGCCCTTGTTCTCGCCCGCGCCG TAGACGTTGGC	
AKRtyl-H130A-F	CATCGACCTCTACCAGTTCgcCCA CGTCGACCGG	Site-directed mutagenesis of AKRtyl- H130A
AKRtyl-H130A-R	AGTGTCCCGGTCGACGTGGgcGA ACTGGTAGAGG	
AKRtyl-E193A-F	TACAACCTCTGCGCGCGCCGCGC CGAGATG	Site-directed mutagenesis of AKRtyl- E193A
AKRtyl-E193A-F	CTCGGCGCGGCGCGCGCAGAGG TTGTACAG	
AKRtyl-E193W-F	TACAACCTCTGCTGGCGCCGCGC CGAGATG	Site-directed mutagenesis of AKRtyl- E193W
AKRtyl-E193W-R	TCGGCGCGGCGCCAGCAGAGGT TGTACAGG	
AKRtyl-E193Q-F	TACAACCTCTGCCAGCGCCGCGC CGAGATG	Site-directed mutagenesis of AKRtyl- E193Q
AKRtyl-E193Q-R	TCGGCGCGGCGCTGGCAGAGGT TGTACAGGC	
AKRtyl-R195A-F	CTCTGCGAGCGCGCCGCCGAGA TGGAGGTC	Site-directed mutagenesis of AKRtyl- R195A
AKRtyl-R195A-R	TCCATCTCGGCGGCGCGCTCGCA GAGGTTG	
AKRtyl-R195W-F	TCTGCGAGCGCTGGGCCGAGAT GGAGGTCG	Site-directed mutagenesis of AKRtyl- R195W
AKRtyl-R195W-R	TCCATCTCGGCCCAGCGCTCGCA GAGGTTG	

AKRtyl-R195K-F	TCTGCGAGCGCAAAGCCGAGATGGAGGTCG	Site-directed mutagenesis of
AKRtyl-R195K-R	TCCATCTCGGCTTTGCGCTCGCAGAGGTTG	AKRtyl- R195K
AKRtyl-S239A-F	GGCAACCGCCGCGCCGCCgCCGGCCGGGCCGCCGAC	Site-directed mutagenesis of
AKRtyl-S239A-R	GCGTCGGCGGCCCGGCCGGcGGCGGCGCGGCGGTTG	AKRtyl- S239A
AKRtyl-S241A-F	GCCGCGCCGCCTCCGGCgcGGCCGCCGACGCGCTC	Site-directed mutagenesis of
AKRtyl-S241A-R	TTGAGCGCGTCGGCGGCCgcGCCGGAGGCGGCGCG	AKRtyl- S241A
AKRtyl-Q250A-F	ACGCGCTCAAGGACCCGGCGCAGCGCGAGCAGATC	Site-directed mutagenesis of
AKRtyl-Q250A-R	TGGATCTGCTCGCGCTGCGCCGGGTCCTTGAGCGC	AKRtyl- Q250A
AKRtyl-Q254W-F	CAGCAGCGCGAGTGGATCCAGCGATACGAGG	Site-directed mutagenesis of
AKRtyl-Q254W-R	TATCGCTGGATCCACTCGCGCTGCTGCGGGTC	AKRtyl- Q254W
AKRtyl-R257A-F	GCGAGCAGATCCAGGCATACGAGGACCTGCTCG	Site-directed mutagenesis of
AKRtyl-R257A-R	CAGGTCCTCGTATGCCTGGATCTGCTCGCGCTG	AKRtyl- R257A
AKRtyl-R257W-F	GAGCAGATCCAGTGGTACGAGGACCTGCTCGAC	Site-directed mutagenesis of
AKRtyl-R257W-R	CAGGTCCTCGTACCACTGGATCTGCTCGCGCTG	AKRtyl- R257W
AKRtyl-W331A-F	CCGGAGGCATTCGCCgcGTGActcgagcaccaccac	Site-directed mutagenesis of
AKRtyl-W331A-R	gtggtgctcgagTCACgcGGCGAATGCCTCCGGTGAC	AKRtyl- W331A

Supplementary References

1. Wheeler, R. et al. Discovery of a Cryptic Intermediate in Late Steps of Mithramycin Biosynthesis. *Angew. Chem. Int. Ed.* **59**, 826-832 (2020).
2. Gulbis, J.M., Mann, S. & MacKinnon, R. Structure of a voltage-dependent K+ channel beta subunit. *Cell* **97**, 943-952 (1999).
3. Gulbis, J.M., Zhou, M., Mann, S. & MacKinnon, R. Structure of the cytoplasmic beta subunit-T1 assembly of voltage-dependent K+ channels. *Science* **289**, 123-127 (2000).
4. Laskowski, R.A. & Swindells, M.B. LigPlot+: Multiple Ligand-Protein Interaction Diagrams for Drug Discovery. *J. Chem. Inf. Model* **51**, 2778-2786 (2011).
5. Laskowski, R.A. PDBsum new things. *Nucleic Acids Res.* **37**, D355-D359 (2009).
6. Flett, F., Mersinias, V. & Smith, C.P. High efficiency intergeneric conjugal transfer of plasmid DNA from *Escherichia coli* to methyl DNA-restricting *streptomycetes*. *FEMS Microbiol. Lett.* **155**, 223-229 (1997).
7. Sun, Y.H., He, X.Y., Liang, J.D., Zhou, X.F. & Deng, Z.X. Analysis of functions in plasmid pHZ1358 influencing its genetic and structural stability in *Streptomyces lividans* 1326. *Appl. Microbiol. Biot.* **82**, 303-310 (2009).
