# Supplementary material for: A three-level regulatory mechanism of the aldo-keto reductase subfamily AKR12D
Source: Nat Commun. 2024 Mar 8;15:2128. doi: 10.1038/s41467-024-46363-z (PMC10923870; doi:10.1038/s41467-024-46363-z)
Supplement: Supplementary file 3 — Description of Additional Supplementary Files [file 41467_2024_46363_MOESM3_ESM.pdf]

Description of Additional Supplementary Files

File Name: Supplementary Data 1
Description: The optimized structure and Gaussian input files of NADPH and tylosin.

File Name: Supplementary Data 2
Description: The initial structures and representative conformations of the trajectories.
