# Supplementary material for: A three-level regulatory mechanism of the aldo-keto reductase subfamily AKR12D
Source: Nat Commun. 2024 Mar 8;15:2128. doi: 10.1038/s41467-024-46363-z (PMC10923870; doi:10.1038/s41467-024-46363-z)
Supplement: Supplementary file 6 — Reporting Summary [file 41467_2024_46363_MOESM6_ESM.pdf]

nature portfolio

Corresponding author(s): Shuangjun Lin, Shaobo Dai and Jian Zhang

Last updated by author(s): Feb 17, 2024

# Reporting Summary

Nature Portfolio wishes to improve the reproducibility of the work that we publish. This form provides structure for consistency and transparency in reporting. For further information on Nature Portfolio policies, see our Editorial Policies and the Editorial Policy Checklist.

## Statistics

For all statistical analyses, confirm that the following items are present in the figure legend, table legend, main text, or Methods section.

| n/a | Confirmed | |
|---|---|---|
| [ ] | [x] | The exact sample size ($n$) for each experimental group/condition, given as a discrete number and unit of measurement |
| [ ] | [x] | A statement on whether measurements were taken from distinct samples or whether the same sample was measured repeatedly |
| [x] | [ ] | The statistical test(s) used AND whether they are one- or two-sided<br>*Only common tests should be described solely by name; describe more complex techniques in the Methods section.* |
| [x] | [ ] | A description of all covariates tested |
| [x] | [ ] | A description of any assumptions or corrections, such as tests of normality and adjustment for multiple comparisons |
| [ ] | [x] | A full description of the statistical parameters including central tendency (e.g. means) or other basic estimates (e.g. regression coefficient) AND variation (e.g. standard deviation) or associated estimates of uncertainty (e.g. confidence intervals) |
| [x] | [ ] | For null hypothesis testing, the test statistic (e.g. $F$, $t$, $r$) with confidence intervals, effect sizes, degrees of freedom and $P$ value noted<br>*Give P values as exact values whenever suitable.* |
| [x] | [ ] | For Bayesian analysis, information on the choice of priors and Markov chain Monte Carlo settings |
| [x] | [ ] | For hierarchical and complex designs, identification of the appropriate level for tests and full reporting of outcomes |
| [x] | [ ] | Estimates of effect sizes (e.g. Cohen's $d$, Pearson's $r$), indicating how they were calculated |

*Our web collection on statistics for biologists contains articles on many of the points above.*

## Software and code

Policy information about availability of computer code

| | |
|---|---|
| Data collection | Crystal diffraction data sets of AKRtyl and AKRtyl complexes were collected at the BL18U1 and BL19U1 beamlines of the Shanghai Synchrotron Radiation Facility by using a DECTRIS PILATUS3 6M detector at a wavelength of 0.97915 Å and 0.97853 Å at 100 K. Kinetic and spectroscopic data were collected using a BioTek Synergy H1 microplate reader and a SX20 stopped-flow spectrometer (Applied Photophysics Ltd., UK). Molecular dynamics simulations and data collection were performed using the AMBER20 package. |
| Data analysis | Diffraction data were processed and scaled using the HKL3000 or HKL2000 program. Structures of AKRtyl and AKRtyl complexes were determined by molecular replacement using the program Phaser (version 2.7.17) from PHENIX(version 1.17.1-3660). Iterative cycles of model rebuilding and refinement were performed using COOT (version 0.9.4) and PHENIX to generate the final model of AKRtyl.<br>Other data processing and figures generation by using Open-Source Pymol (Schrödinger), GraphPad Prism 8.0.1, BioTek Gen5 3.05.11, AppliedPhotophysics Pro-Data Viewer 4.4.2.0, Qualitative Analysis Navigator B.08.00, EFI-EST (https://efi.igb.illinois.edu), Cytoscape 3.9.1, AMBER20, VMD 1.9.1, ProDy 2.0, MEGA 11, ChiPlot (https://www.chiplot.online/#Phylogenetic-Tree), Clustal Omega (https://www.ebi.ac.uk/Tools/msa/clustalo) and ESPript 3.0 (https://espript.ibcp.fr/ESPript/cgi-bin/ESPript.cgi). |

For manuscripts utilizing custom algorithms or software that are central to the research but not yet described in published literature, software must be made available to editors and reviewers. We strongly encourage code deposition in a community repository (e.g. GitHub). See the Nature Portfolio guidelines for submitting code & software for further information.

## Data

Policy information about availability of data

All manuscripts must include a data availability statement. This statement should provide the following information, where applicable:

- Accession codes, unique identifiers, or web links for publicly available datasets
- A description of any restrictions on data availability
- For clinical datasets or third party data, please ensure that the statement adheres to our policy

Data supporting the findings of this work are available within the paper and its Supplementary Information/Source Data files. X-ray crystallographic coordinates have been deposited in the Protein Data Bank (PDB) with the accession codes: 8XR2 [https://www.rcsb.org/structure/8XR2], 8XR3 [https://www.rcsb.org/structure/8XR3], 8JWL [https://www.rcsb.org/structure/8JWL], 8JWK [https://www.rcsb.org/structure/8JWK], 8XR4 [https://www.rcsb.org/structure/8XR4], 8JWN [https://www.rcsb.org/structure/8JWN], 8JWM [https://www.rcsb.org/structure/8JWM], 8JWO [https://www.rcsb.org/structure/8JWO].

## Research involving human participants, their data, or biological material

Policy information about studies with human participants or human data. See also policy information about sex, gender (identity/presentation), and sexual orientation and race, ethnicity and racism.

| | |
|---|---|
| Reporting on sex and gender | n/a |
| Reporting on race, ethnicity, or other socially relevant groupings | n/a |
| Population characteristics | n/a |
| Recruitment | n/a |
| Ethics oversight | n/a |

Note that full information on the approval of the study protocol must also be provided in the manuscript.

# Field-specific reporting

Please select the one below that is the best fit for your research. If you are not sure, read the appropriate sections before making your selection.

[x] Life sciences  [ ] Behavioural & social sciences  [ ] Ecological, evolutionary & environmental sciences

For a reference copy of the document with all sections, see nature.com/documents/nr-reporting-summary-flat.pdf

# Life sciences study design

All studies must disclose on these points even when the disclosure is negative.

| | |
|---|---|
| Sample size | No statistical method was used to predetermine sample size in this work. Following common practice, the enzyme activities of wild type and each mutants were independently determined in triplicate or more. |
| Data exclusions | No data exclusions. |
| Replication | All experiments were performed in triplicate or more and all attempts at replication were successful. |
| Randomization | Sample were not allocated into experimental groups and thus no randomization is needed for these assays. This aspect of study design is not relevant. |
| Blinding | Blinding is not necessary for this research. Appropriate control experiments were included. |

# Reporting for specific materials, systems and methods

We require information from authors about some types of materials, experimental systems and methods used in many studies. Here, indicate whether each material, system or method listed is relevant to your study. If you are not sure if a list item applies to your research, read the appropriate section before selecting a response.

## Materials & experimental systems

| n/a | Involved in the study |
| --- | --- |
| x | Antibodies |
| x | Eukaryotic cell lines |
| x | Palaeontology and archaeology |
| x | Animals and other organisms |
| x | Clinical data |
| x | Dual use research of concern |
| x | Plants |

## Methods

| n/a | Involved in the study |
| --- | --- |
| x | ChIP-seq |
| x | Flow cytometry |
| x | MRI-based neuroimaging |
